# Widespread energy limitation to life in global subseafloor sediments

**DOI:** 10.1126/sciadv.aba0697

**Published:** 2020-08-05

**Authors:** J. A. Bradley, S. Arndt, J. P. Amend, E. Burwicz, A. W. Dale, M. Egger, D. E. LaRowe

**Affiliations:** 1 School of Geography, Queen Mary University of London, London, UK.; 2 Department of Geochemistry, GFZ, German Research Centre for Geosciences, Potsdam, Germany.; 3 Department of Geosciences, Environment and Society, Université Libre de Bruxelles, Brussels, Belgium.; 4 Department of Earth Sciences, University of Southern California, Los Angeles, CA, USA.; 5 Department of Biological Sciences, University of Southern California, Los Angeles, CA, USA.; 6 GEOMAR, Helmholtz Centre for Ocean Research, Kiel, Germany.; 7 The Ocean Cleanup Foundation, Rotterdam, Netherlands.

## Abstract

Microbial cells buried in subseafloor sediments comprise a substantial portion of Earth’s biosphere and control global biogeochemical cycles; however, the rate at which they use energy (i.e., power) is virtually unknown. Here, we quantify organic matter degradation and calculate the power utilization of microbial cells throughout Earth’s Quaternary-age subseafloor sediments. Aerobic respiration, sulfate reduction, and methanogenesis mediate 6.9, 64.5, and 28.6% of global subseafloor organic matter degradation, respectively. The total power utilization of the subseafloor sediment biosphere is 37.3 gigawatts, less than 0.1% of the power produced in the marine photic zone. Aerobic heterotrophs use the largest share of global power (54.5%) with a median power utilization of 2.23 × 10^−18^ watts per cell, while sulfate reducers and methanogens use 1.08 × 10^−19^ and 1.50 × 10^−20^ watts per cell, respectively. Most subseafloor cells subsist at energy fluxes lower than have previously been shown to support life, calling into question the power limit to life.

## Teaser

The global subseafloor biosphere subsists under extreme and widespread energy limitation.

## INTRODUCTION

Marine sediments harbor a vast number of microorganisms across a wide range of depositional settings (*1*). These microorganisms have been shown to survive for extraordinarily long periods of time (*2*). Contrary to life at Earth’s surface, subsurface organisms are severely energy limited (*3*). Many of these sediment-dwelling microbes are novel and uncultured (*4*, *5*) and survive under extreme energy limitation for millennia, thus calling into question the limit for life (*2*, *6*). They are also responsible for degrading Earth’s largest pool of organic C (*7*), exerting a major control on global climate and biogeochemical cycles (*8*). The availability of energy to microorganisms in subseafloor sediments varies considerably from one environment to another (*9*) and imposes limits on their rates of activity, as well as their survival (*3*, *10*). However, outside of measurements from specific sites (*11*–*13*) and laboratory experiments (*14*), the rate of energy utilization, i.e., power, of subseafloor life is not well known. Therefore, questions pertaining to microbial activity levels, community assembly, adaptive evolution, and even the lower power limit to life remain unanswered (*3*, *15*, *16*). Here, we quantify the power utilization for all microorganisms contained in subseafloor sediments that were deposited during the past 2.59 million years (i.e., the Quaternary period). A bioenergetic ecosystem model was developed and implemented on a 0.25° × 0.25° resolution global grid. Our model calculates the volumetric distribution, rate, and thermodynamic properties of particulate organic carbon (POC) degradation, as well as the global distribution of cells and electron acceptors, in Quaternary-age subseafloor sediments. On this basis, we calculate, in three dimensions, the cell-specific power of microbial life in the global marine sediment habitat.

## RESULTS AND DISCUSSION

### Metabolism and organic carbon degradation in global marine sediments

**Fig. 1:**
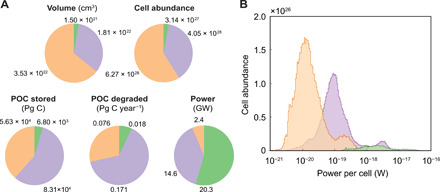
Volumetric distribution and flux of sediment, cells, organic carbon, and energy across major marine sediment catabolic zones in global Quaternary subseafloor sediment. (**A**) The proportion of total sediment volume (cubic centimeters), number of microbial cells, POC stored (petagrams of C) and degraded (petagrams of C per year), and power used (gigawatts) in each catabolic zone in Quaternary sediment. (**B**) Frequency distribution of cell-specific power utilization in Quaternary-age sediment. Green denotes oxic, lilac signifies sulfate-reducing, and orange represents methanogenic sediment.

We designate POC degraded in oxic sediments to be mediated by O_2_, POC degraded in the sulfate-reducing zone to be mediated by SO_4_^2−^, and attribute POC degraded beneath the sulfate-methane transition to methanogenesis (reactions 1 to 3; table S1). The thickness and depth of Holocene- and Pleistocene-aged sediments, as well as the depth of oxygen penetration and the depth of the sulfate-methane transition, are highly variable across the seafloor (fig. S1). Oxic sediments are mostly restricted to deep-water abyssal zones (fig. S2). Together, we estimate that 2.7% of the Quaternary subseafloor sediment volume is oxic and that it contains 6.80 × 10^3^ Pg of organic C (4.6% of Quaternary subseafloor sediment POC). Our mechanistically derived estimate for the global mass of organic carbon in oxic marine sediment (6.80 × 10^3^ Pg of C) is very closely aligned with an independent empirically derived estimate (6.7 × 10^3^ to 16.0 × 10^3^ Pg of C) (*17*). Sulfate-reducing and methanogenic sediments are more widespread (33.0 and 64.3% of sediment volume, respectively), particularly underlying shallow-water shelf and margin settings, and in deeper and thicker sedimentary layers (fig. S2). We calculate that sulfate-reducing sediments contain 8.31 × 10^4^ Pg of organic C (56.9% of Quaternary subseafloor sediment POC) and methanogenic sediments contain 5.63 × 10^4^ Pg of organic C (38.5% of Quaternary subseafloor sediment POC). We find that rates of POC degradation are mostly reflective of the volumetric POC distribution across subseafloor sediments. The majority of POC degradation in Quaternary sediments globally is mediated by sulfate reduction (64.5%; 0.171 Pg of C year^−1^). We calculate that oxic sediments mediate 6.9% of global subseafloor POC degradation (0.018 Pg of C year^−1^), while methanogenic sediments mediate 28.6% (0.076 Pg of C year^−1^) (Fig. 1). Our results underpin the understanding that sulfate plays a major role in the global degradation of organic C in marine sediments (*18*–*20*), while simultaneously verifying the importance of methanogenesis (*21*).

### Power utilization in global marine sediments

The flux of energy, i.e., power, that is supplied to and used by microorganisms is calculated using the rates of POC degradation combined with the Gibbs energies of O_2_, SO_4_^2−^, and CH_4_-mediated catabolism under representative in situ conditions (tables S1 and S2). The combined total power used by all microorganisms in subseafloor sediment is 37.3 GW. This is equivalent to only 0.08% of the power produced photosynthetically in the marine photic zone (*22*, *23*). Microbial power utilization is the highest, per cubic centimeter of sediment, in shallow coastal settings (up to 10^−9^ W cm^−3^), and it is uniformly low (~10^−14^ W cm^−3^) in the oldest Pleistocene sediments (2.59 million years) (fig. S2). High power (per cubic centimeter) in sediments underlying northern high latitudes and Southeast Asian shelves can be attributed to high concentrations of reactive POC (fig. S3) that degrade relatively rapidly.

Notably, we find that O_2_-mediated POC degradation constitutes the major power source in subseafloor sediments (20.3 GW; 54.5% of global subseafloor power), despite oxic sediment comprising a minor fraction of total sediment volume and POC. Microorganisms in sulfate-reducing sediment use 14.6 GW of power globally (39.1% of the global total), while microorganisms in methanogenic sediments use only 2.4 GW, equivalent to only 6.4% of the global subseafloor power utilization. The dichotomy between both the volume and amount of POC stored within each sedimentary layer and the power realized by POC degradation for each catabolic strategy underscores the need to consider not only POC concentrations and other geochemical constituents but also the rate and energetics of major catabolic pathways occurring in the subseafloor biosphere.

### Cell-specific power utilization

The power per individual microbial cell is calculated by dividing the total power used by the number of microorganisms present in a sedimentary horizon. Our results confirm that subseafloor sediment microorganisms are characterized by extreme and widespread energy limitation. We calculate that 84.0% of microorganisms subsist at an energy flux less than the lowest previously calculated estimate of the basal power limit to life for natural settings [1.9 × 10^−19^ W per cell (*13*)]. The median rate of microbial energy utilization in Quaternary-age marine sediments, i.e., cell-specific power, is 3.32 × 10^−20^ W per cell, which is more than two orders of magnitude lower than the lowest experimentally derived maintenance power for microorganisms (*24*) and five orders of magnitude lower than the lowest culture-based estimates of maintenance power (*25*).

Stepwise decreases in microbial power utilization largely follow the redox state of the sediment (Fig. 1). The delineation of microbial power utilization according to catabolic zones is consistent with a continuous decrease in reactivity and degradation rate of organic matter with increasing sediment depth and sediment age (*26*). Microorganisms situated within oxic sediments exhibit the highest power per cell, with a median power utilization of 2.23 × 10^−18^ W per cell. This is broadly consistent with field data–derived estimates of the maintenance power of microorganisms from oxygen-bearing sediments [5.30 × 10^−18^ W per cell (*6*)]. The power utilization of microorganisms situated in anoxic sediments is generally lower than that in oxic sediments (*27*, *28*). This is clearly reflected by our data: We find that cell-specific power in anoxic sediments is one to two orders of magnitude lower than that in oxic sediments, with a median power utilization of 1.08 × 10^−19^ and 1.50 × 10^−20^ W per cell in sulfate-reducing and methanogenic sediments, respectively. Globally, we find that extremely few cells (<0.02%) subsist at less than 1 zW (10^−21^ W), substantiating a theoretically posed “minimum decay prevention power” for microbial life (*13*).

**Fig. 2:**
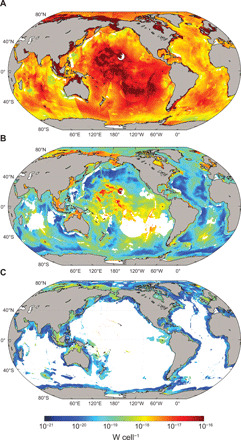
Cell-specific power utilization across major marine sediment catabolic zones. Power per cell (watts) calculated on a global scale and depth-integrated for the (**A**) oxic, (**B**) sulfate-reducing, and (**C**) methanogenic sedimentary layers. White areas denote absence of the corresponding catabolic zone.

Distinct high- and low-power zones arise according to catabolic strategy and geography (Fig. 2). It can be shown that the somewhat multimodal distribution of cellular power utilization (Fig. 1) arises from log-normal distributions within each domain (i.e., shelf, margin, and abyssal settings) (fig. S4). Microorganisms contained in continental shelf sediments, particularly in northern high latitudes and near Southeast Asia, use comparatively higher power than elsewhere (Fig. 2). This highlights that sediment POC with relatively short depositional pathways (i.e., situated in shallow water, <200 m) is tilted toward higher reactivity (fig. S3) (*29*) and supports microorganisms at a higher activity state (Fig. 2). High cell-specific power is also prevalent in oxic sediments underlying the ocean rises of the East and Central Pacific, the Southwest and Southeast Indian Ridges, and the Mid-Atlantic Ridge (2000 to 3000 m). These regions are characterized by low cell abundance (*1*, *2*), where microorganisms are sustained for millennia with extremely low mortality (*2*, *30*) on ancient POC (*17*), but via a highly exergonic terminal electron transfer step (table S2). Sulfate-reducing sediments underlie many of the major ocean basins (>4000-m water depth), including the North Pacific, North Atlantic, Argentine, Canary, Angola, Cape, Southwest Pacific, and Mid–Indian Ocean basins. These sediments contain cells using very little power (<10^−20^ W per cell). Methanogenesis, which is prevalent in shallow-water sediments near continental land masses, is shown to provide even less power to individual organisms (10^−21^ to 10^−20^ W per cell). These extremely low-power settings, largely in deeper and thicker sedimentary layers underlying shallow-water shelf and margin settings, are typically characterized by high rates of mortality among subseafloor microbial communities (*31*). Nevertheless, the sustained presence of microbes, even in deeper, low-power sedimentary layers, indicates that at least a fraction of the microbial population are capable of surviving at powers far lower than has been calculated for any natural environment previously (*13*).

Uncertainty in our calculations arises from (i) the rate of POC degradation, (ii) the Gibbs energy of the reaction, and (iii) the number of cells carrying out the reaction. Uncertainty is evaluated for each of these factors (i to iii) individually and concurrently (i.e., together) (see Supplementary Text). The reactivity of POC deposited onto the seafloor and its evolution during burial are accounted for by a set of adjustable parameters (see Materials and Methods) using a theoretically and empirically well-established reactive continuum modeling approach, validated using a large body of carefully dated organic matter and pore water depth profiles, rate measurements, isotopic signatures, and authigenic mineral distributions from a wide range of marine settings across contrasting depositional environments and temporal scales. We prescribe parameter values that are consistent with decades of observations, laboratory experiments, and modeling, in which the decrease in organic matter reactivity with increasing sediment depth and age, as well as from continental to abyssal environments, is widely observed (*29*). Nevertheless, to address the uncertainty in our results associated with (i) POC degradation rates, we evaluate a range of simulations encapsulating all possible variability in the reactivity of POC deposited onto the seafloor and its evolution during burial, constrained by a global parameter compilation (see Supplementary Text). To address the uncertainty associated with variability in (ii) the Gibbs energy arising from spatial heterogeneities in the concentrations of reactants and products, we run additional simulations, where the concentrations of the reactants and products are varied over realistic ranges to represent high-energy and low-energy scenarios. The calculation of power per cell also relies on an estimate of the number of cells carrying out the reaction, for which we use the best available spatially resolved global model for subseafloor biomass distribution (*1*) and simplify the reaction network to account for the most pertinent biogeochemical processes and the major catabolic pathways of POC oxidation. A large body of evidence suggests that the vast majority of microorganisms in subseafloor sediments are involved in the degradation of organic matter and overwhelmingly hold the genetic potential to degrade organic molecules, especially in organic-rich, coastal, and/or anoxic settings (see Supplementary Text). Nevertheless, to account for uncertainty in (iii) the number of microbial cells present in the sediment and carrying out the metabolism prescribed to a certain redox zone, we carry out additional simulations where cell abundance is varied by up to 50%. These factors (i to iii), tested individually and simultaneously, produce relatively minor deviations in the median-per-cell power (1.42 × 10^−20^ to 10.20 × 10^−20^ W per cell; see Supplementary Text). Under all tested scenarios, the percentage of microorganisms subsisting at less than 1.9 × 10^−19^ W per cell is at least 65.7%—underscoring the highly energy-limited nature of microbial life in subseafloor sediments.

Quantifying the energy turnover of the subseafloor biosphere is critical to determining the cell-specific minimum power requirement for life, as well as to predict the habitable boundaries of life on Earth. This study advances previous knowledge of the abundance, distribution, and activity of microorganisms in the subsurface (*1*, *32*, *33*) by providing the catabolic rates and specific activities of these microorganisms on a global scale. Considering that a small number of cells use a disproportionately large fraction of the total energy in marine sediments, we find that extraordinarily low power is a characteristic of almost all subseafloor sediment–hosted life. We report energy fluxes that fall short of previously calculated cell maintenance requirements for natural settings, verifying the overwhelmingly low-power nature of the deep biosphere (*10*). Considering that cells are subsisting at or near basal maintenance power requirements (*13*), it is unlikely that growth (i.e., cellular division), and by extension evolution, can be sustained widely throughout subseafloor sediments, validating what has shown to be the case for specific sites (*15*). The prevalence of dormant and nongrowing microorganisms, however, affords a survival advantage permitting ecological coexistence (*34*) and maintenance of biodiversity (*35*) in subseafloor sediments. Overall, our data represent evidence for widespread energy limitation in the marine subsurface, and that the overwhelming majority of subseafloor sediment microorganisms may subsist at close to the power limit for life.

## MATERIALS AND METHODS

### Experimental design

Here, we present and apply a new bioenergetic modeling approach that is used to quantify the distribution and rate of POC degradation, as well as the global distribution of cells, electron acceptors, and the thermodynamic properties of POC degradation. On this basis, we calculate the cell-specific power (i.e., energy per unit time per individual microbial cell), *P* (watts) according to

$$P=\frac{r\cdot \Delta G_{r}}{Y}$$ (1)

where *r* denotes the rate of reaction (grams of C degraded per cubic centimeter per second), Δ*G*_r_ (joules per gram of C) represents the Gibbs energy of the reaction, and *Y* (cells per cubic centimeter) is the number of microbial cells carrying out the reaction. The modeling framework is implemented on a 0.25° × 0.25° resolution global grid using the following global datasets and models.

### Reaction-transport model

We use a one-dimensional reaction-transport model (RTM) to calculate the distribution and degradation rate of POC in subseafloor sediments throughout the Quaternary period, following the approach described in (*36*). Similar to Wallmann *et al.* (*37*), constant boundary conditions and parameters are used to characterize the Holocene (0 to 11,700 years old) and Pleistocene (11,700 years old to 2.59 million years old) depositional environments.

The one-dimensional conservation equation for POC in porous media is given by (*38*–*39*)

$$\begin{array}{c} \frac{\partial (1-\Phi )\text{POC}}{\partial t}=\frac{\partial }{\partial z}(D_{b}(1-\Phi )\frac{\partial \mathrm{POC}}{\partial z})-\\ \frac{\partial (1-\Phi )\omega \text{POC}}{\partial z}+(1-\Phi )R_{\text{POC}} \end{array}$$ (2)

where POC (grams of C per cubic centimeter dry sediment) is the concentration of POC, *t* refers to time, *D*_b_ (square centimeters per year) stands for the bioturbation coefficient, ω (centimeters per year) represents the sedimentation rate, and *R*_POC_ (grams of C per cubic centimeter per year) denotes the rate of organic matter degradation. The porosity, Φ, of marine sediments in the shelf, margin, and abyss domains was calculated as a function of depth, *z* (meters), assuming steady-state compaction, according to (*40*)

$$\Phi_{z}=\Phi_{0}\cdot e^{-c_{0}\cdot z}$$ (3)

where Φ_0_ denotes the porosity at the sediment-water interface (SWI) and *c*_0_ (per meter) stands for the compaction length scale, which characterizes how a given sediment type will compact under its own weight.

Quaternary sediments are divided into three layers: Holocene bioturbated [top 10 cm (*39*)], nonbioturbated Holocene (<11,700 years), and Pleistocene (<2.59 million years). Sediment mixing was assumed to be constant over the bioturbated layer and nonexistent immediately below it. The rate of organic matter degradation, *R*_POC_, was described using a reactive continuum model (RCM). The RCM assumes a continuous yet dynamic distribution of organic compounds comprising a range of reactivities and reproducing the often-observed decrease in apparent POC reactivity with increasing sediment depth (and thus age) (*41*). Within the RCM, *R*_POC_ is given by

$$R_{\text{POC}}=-\int_{0}^{\infty }\;k\cdot \mathrm{om}(k,t)\mathrm{dk}$$ (4)

where *om*(*k*,*t*) represents a probability density function that determines the concentration of organic matter having a degradability between *k* and *k* + *dk* at time *t*, with *k* being analogous to a reaction rate constant. The initial distribution or organic compounds [*om*(*k*,*0*)] cannot be inferred by observations and may take different mathematical forms. We use a gamma function (*36*, *41*–*43*), assuming first-order degradation kinetics, whereby the initial (*t* = 0) distribution of *om* over *k* is given by

$$\mathrm{om}(k,0)=\frac{{\text{POC}}_{0}\cdot a^{\nu }\cdot k^{\nu -1}\cdot e^{-a\cdot k}}{\Gamma (\nu )}$$ (5)

where POC_0_ is the initial organic matter content (at the SWI), Γ is the gamma function, *a* (years) is the average lifetime of the reactive components of the POC, and ν is a dimensionless parameter determining the shape of the distribution near *k* = 0. Assuming steady-state conditions (∂POC/∂*t* = 0) and a known organic C content at the sediment water interface, POC_0_, the change in the POC concentration as a function of depth, POC(*z*), is given by (*41*)

$$\text{POC}(z)={\text{POC}}_{0}\cdot {\left(\frac{a}{a+\text{age}(z)}\right)}^{\nu }$$ (6)

where age(*z*) refers to the age of the sediment layer at depth *z*. Following the approaches described in (*36*, *44*), we use a multi-G approximation of the RCM for the bioturbated sediment. Within the bioturbated zone, POC is represented by 500 distinct fractions that are degraded according to a first-order organic matter degradation rate law with a degradation rate constant, *k_i_*

$$R_{\text{POC}}=\sum_{i=1}^{500}k_{i}\cdot {\text{POC}}_{i}(z)$$ (7)

where

$${\text{POC}}_{i}(0)=F_{i}\cdot \text{POC}$$ (8)

The initial proportion of total organic matter in fraction *i*, *F_i_*, as well as its respective reactivity, *k_i_*, can be determined through the initial probability density function that determines the concentration of organic matter having a degradability between *k* and *k* + *dk* at time 0 (Eq. 4). The initial fraction of total POC characterized by a distinct reactivity *k* is given by

$$f(k,0)=\frac{\mathrm{om}(k,0)}{{\text{POC}}_{0}}=\frac{{\mathrm{ia}}^{\nu }\cdot k^{\nu -1}\cdot e^{-a\cdot k}}{\Gamma (\nu )}$$ (9)

The initial fraction of POC within the reactivity range between 0 and *k*, i.e., having a reactivity ≤*k*, is then given by integrating Eq. 9, assuming *a*, ν, and *k* > 0

$$\begin{array}{cc} F(k,0) & =\int_{0}^{k}\;f(0,k)\mathrm{dk}=\int_{0}^{k}\;\frac{a^{\nu }\cdot k^{\nu -1}\cdot e^{-a\cdot k}}{\Gamma (\nu )}\mathrm{dk}\\ & =\frac{a^{\nu }\cdot k^{\nu }\cdot {\left(a\cdot k\right)}^{-v}(\Gamma (\nu )-\Gamma (\nu ,a\cdot k))}{\Gamma (\nu )}\\ & =(\frac{1-\Gamma (\nu ,a\cdot k)}{\Gamma (\nu )}) \end{array}$$ (10)

where Γ(ν,*a*∙*k*) denotes the inverse gamma function.

In the bioturbated sediment, the RCM is approximated by dividing the reactivity range *k* = [10^−15^, 10^(−log(*a*) + 2)^] into 500 equal reactivity bins, *k_j_*, thus ensuring a comprehensive approximation of the gamma function defined by the respective *a* and ν values. The initial fraction, *F_i_*, of total POC within the reactivity bin *k*_*j*−1_ and *k_j_* (and thus with reactivity *k_i_* = *k*_*j*−1_ + (*k_j_* − *k*_*j*−1_)/2) in the 500G model can then be calculated as

$$F_{i}=F(k_{j},0)-F(k_{j-1},0)$$ (11)

The most reactive fraction, *F*_500_, with reactivity *k*_500_ = 10^−log(*a*) + 2^ year^−1^ is calculated on the basis of the upper incomplete gamma function

$$F_{500}=\int_{k_{500}}^{\infty }f(k_{500},0)\mathrm{dk}=\frac{\Gamma (\nu ,a\cdot k_{500})}{\Gamma (\nu )}$$ (12)

The derived rate constants are used in Eq. 2 by expressing *R*_POC_ according to Eq. 7 to determine POC concentrations and degradation rates in the Holocene bioturbated layer (<10 cm). Assuming steady-state conditions, the general solution of Eq. 2 for each organic matter fraction *i* is given by

$${\text{POC}}_{i}(z)=A_{i}e^{\left(\alpha_{i}z\right)}+B_{i}e^{\left(b_{i}z\right)}$$ (13)

where

$$\alpha_{i}=\frac{\omega -\sqrt{\omega^{2}+4D_{b}\cdot k_{i}}}{2D_{b}}$$ (14)

$$b_{i}=\frac{\omega +\sqrt{\omega^{2}+4D_{b}\cdot k_{i}}}{2D_{b}}$$ (15)

and

$$\text{POC}(z)=\sum_{i=1}^{500}\;{\text{POC}}_{i}(z)$$ (16)

The integration constants *A_i_* and *B_i_* are defined by the chosen boundary conditions. Here, we apply a known POC concentration at the sediment-water interface [POC(0) = POC_0_] and assume continuity (equal flux and concentration) across the bottom of the bioturbated layer, i.e.

$$\text{POC}(z\text{bio})={\text{POC}}_{z\text{bio}};{\frac{-D_{b}d\text{POC}}{\mathrm{dz}}|}_{z\text{bio}}=0$$ (17)

Below the Holocene bioturbated zone, the values of age(*z*) that are required to evaluate Eq. 6 are calculated using burial rates, ω(*z*), porosity depth profiles, Φ(*z*), and the apparent age of organic matter at the lower limit of the Holocene bioturbated zone, age_*z*bio_. By inserting POC_bio_ and age_*z*bio_ into Eq. 6 for POC(*z*) and age(*z*), respectively, age_*z*bio_ can be solved

$${\text{age}}_{z\text{bio}}=\frac{-a\cdot (\text{exp}(\text{ln}({\text{POC}}_{\text{bio}}/{\text{POC}}_{0})/\nu )-1)}{\text{exp}(\text{ln}({\text{POC}}_{\text{bio}}/{\text{POC}}_{0})/\nu )}$$ (18)

Assuming an exponentially decreasing porosity, Eq. 2, and steady-state compaction, the burial velocity, ω, at depth *z* is then [e.g., (*38*)]

$$\omega (z)=(\frac{1-\Phi_{0}}{1-\Phi (z)})\omega_{0}$$ (19)

where ω_0_ corresponds to the burial velocity at the SWI. The age of a given sediment layer at depth *z* below the Holocene bioturbated zone, age(*z*), is given by [e.g., (*38*)]

$$\text{age}(z)=\int_{0}^{z}\;\omega^{-1}\mathrm{dz}$$ (20)

Substituting Eq. 19 into Eq. 20 results in

$$\text{age}(z)=\frac{1}{(1-\Phi_{0})\omega_{0}}\;\int_{0}^{z}\;(1-\Phi )\mathrm{dz}$$ (21)

which, upon integration, leads to

$$\text{age}(z)=\frac{z+\frac{\Phi_{0}}{c_{0}}\cdot (\text{exp}(-c_{0}\cdot z)-1)}{\omega_{0}\cdot (1-\Phi_{0})}$$ (22)

The age of POC below the Holocene bioturbated zone is thus given by

$$\text{age}(z)={\text{age}}_{z\text{bio}}+\frac{z+\frac{\Phi_{0}}{c_{0}}\cdot (\text{exp}(-c_{0}\cdot (z-z_{\text{bio}}))-1)}{\omega_{0}\cdot (1-\Phi_{0})}$$ (23)

The depth distribution of organic matter in marine sediments deposited since the beginning of the Quaternary period can thus be calculated with knowledge of the sedimentation rate, level of bioturbation, porosity structure, bulk organic matter concentration at the SWI, and the distribution of organic compounds across the reactivity range at the sediment-water interface.

### Total POC budget

The fraction of total POC preserved in a layer of sediment that accumulated over a given time interval, $\bar{{\text{PE}}_{i}}$, is given as the ratio of the total amount of POC stored in the *i*th sediment layer [*i* = Holocene bioturbated (0 to *z*_bio_), Holocene nonbioturbated (*z*_bio_ to *z*_holo_), and Pleistocene (*z*_holo_ to *z*_pleis_)], $\bar{{\text{POC}}_{i}}$ (grams of C per square centimeter), and the total steady-state input of POC to that respective layer, $\bar{I_{i}}$, (grams of C per square centimeter)

$$\bar{{\text{PE}}_{i}}=\bar{{\text{POC}}_{i}}/\bar{I_{i}}$$ (24)

where

$$\bar{{\text{POC}}_{i}}=\int_{z_{i}}^{z_{i-1}}\text{POC}(z)\mathrm{dz}$$ (25)

and

$$\bar{I_{i}}=\text{POC}(z_{i}-1)\cdot \Delta z_{i}$$ (26)

The amount of POC degraded in the *i*th layer, $\bar{R_{i}}$, is given by

$$\bar{R_{i}}=\int_{z_{i}}^{z_{i-1}}k(z)\cdot \text{POC}(z)\mathrm{dz}$$ (27)

Two different global datasets (0.25° × 0.25°) of values for ω, the sedimentation rate, are used: one for Holocene bioturbated and Holocene sediments and the other for Pleistocene sediments.

### Parameters and forcings

The concentration of Holocene and Pleistocene POC at the sediment-water interface, POC_0_, and sedimentation rates, ω, are constrained according to (*37*), which used data from (*45*, *46*) and an algorithm that correlates water depth and sedimentation rate (*47*). Following (*36*), the bioturbation coefficient is calculated as a function of water depth based on a compilation of empirical data collected in (*48*). Its values range spatially from 0.59 to 27 cm^2^ year^−1^, decreasing in magnitude as water depth increases. It is constant throughout the depth of Holocene bioturbated zone and immediately drops to zero beneath it. Values of Φ_0_ and *c*_0_ are chosen to describe the shelf, margin, and abyss based on sediments that are representative of these domains (see table S3) (*49*). We partitioned the ocean floor into shelf, margin, and abyss domains to specify values for some of the model parameters (fig. S5 and table S3). We choose a constant ν parameter of 0.125 for all three sediment domains, which is characteristic of fresh organic matter and based on observations that the ν parameter values do not vary much between sites (*29*, *41*). We link values of the *a* parameter with sedimentation rates, based on global observations (*29*, *41*). This approach accounts for order-of-magnitude changes in *a* due to factors that control organic matter transit times from its source to deposition. These parameters thus reflect typically observed RCM parameter variability across various depositional environments. Uncertainties arising from parameter values are addressed in Supplementary Text.

### Global reaction network and catabolic strategy

We designate all sediments shallower than the maximum O_2_ penetration depth as oxic. To map O_2_ penetration depth in marine sediments on a global scale (fig. S1), we combine (i) global datasets of oxygen concentrations in marine sediments and (ii) previous modeling results (*2*). First, we use a global dataset of oxygen concentrations and corresponding water depths (table S4) to formulate a regression model [*R*^2^ = 0.64; based on (*50*)]. This model is extrapolated over the entire ocean floor. For data in columns 81 to 91 (table S4), oxygen penetration depth was determined following the methods described in (*51*). Second, we superimpose (onto our global map) the results from a modeling study (*2*), which designated regions of the seafloor where O_2_ is known to penetrate through all sedimentary layers to basement rock. We define the sulfate reduction zone as the horizon between the maximum O_2_ penetration depth and the depth of the sulfate-methane transition. We use data from (*52*) to map the global sulfate-methane transition depth onto our 0.25° × 0.25° global grid. The reaction zones designated within our model are based on the best available published datasets and include not only global compilations of marine sediment core data (*50*, *52*) but also global models (*2*, *52*). Data from the numerous core samples (*n* = 1704) documenting the distribution of sulfate-reducing and methanogenic sediments included in our study are available via (*52*) and the International Ocean Discovery Program. We designate POC degraded in oxic sediments to be mediated by O_2_, POC degraded in the sulfate-reducing zone to be mediated by SO_4_^2−^, and prescribe POC degraded beneath the sulfate-methane transition to methanogenesis (reactions 1 to 3; table S1), thus simplifying the reaction network to account for the most pertinent biogeochemical process, based on widely accepted modeling and experimental studies (*18*, *21*, *53*). Although other oxidants are used by microorganisms for degrading organic matter in marine sediments, such as nitrate, and metal oxides, these have been shown to be quantitatively unimportant metabolic pathways on a global scale (*29*, *44*, *54*–*56*)—mainly because of their low concentration compared to SO_4_^2−^, the additional consumption of Fe and Mn oxides in a range of rapid secondary redox reactions (*57*), and the rapid exchange of O_2_ in shallow sediment. O_2_, SO_4_^2−^, and CH_4_-mediated POC degradation are considered here because the vast majority of marine sedimentary organic carbon is degraded via these pathways (*18*, *21*, *54*). Energy may also be provided by fermentation, although its importance is not well known. We use acetate (CH_3_COO^−^) as a proxy for POC. To undertake a study of this scale, such an assumption is essential, since the identity of the thousands of organic molecules consumed by microorganisms in natural settings is spatially disparate and is rarely known. Acetate is a fair choice of molecule, being a regular constituent of marine sediment pore water (*26*). Fractional changes in Gibbs energies associated with the choice of organic compound do not meaningfully affect our results (see “Uncertainty” section in Supplementary Text). In addition, the Gibbs energies of organic matter degradation are, on a per-electron basis, much more sensitive to the identity of the electron acceptor than that of the organic compound (*58*, *59*). By focusing on the oxidant, we are capturing the first-order energetic differences of organic matter degradation in different environmental settings.

### Gibbs energy calculations

The amount of energy available from the oxidation of organic matter by aerobic, sulfate-reducing, and methanogenic pathways is calculated on the basis of the Gibbs energy function

$$\Delta G_{r}=\Delta G_{r}^{0}+\mathrm{RT}\;\text{ln}Q_{r}$$ (28)

where Δ*G*_r_^0^ and *Q*_r_ refer to the standard molal Gibbs energy and the reaction quotient of the indicated reaction, respectively, *R* represents the gas constant, and *T* denotes temperature in kelvin. Values of Δ*G*_r_^0^ were calculated using the revised Helgeson-Kirkham-Flowers equations of state (*60*–*62*), the SUPCRT92 software package (*63*), and thermodynamic data taken from (*64*–*68*). Individual values of *Q*_r_ are calculated for each reaction using

$$Q_{r}=\prod a_{i}^{v_{i}}$$ (29)

where *a_i_* stands for the activity of the *i*th species and *v_i_* corresponds to the stoichiometric coefficient of the *i*th species in the reaction of interest. Activity of the *i*th species is calculated according to

$$a_{i}=m_{i}\gamma_{i}$$ (30)

where *m_i_* and γ*_i_* denote the molality and individual activity coefficient of the *i*th species, respectively. Values of γ*_i_* are computed as a function of temperature and ionic strength using an extended version of the Debye-Hückel equation (*69*).

We have selected specific sets of representative conditions that are used to calculate Gibbs energies of each reaction (table S2). We define reactions at 5°C and 100 bars of pressure. For aerobic heterotrophy, we base our calculations on chemical data from oxic South Pacific Gyre sediments (*2*). For sulfate-reducing and methanogenic sediments, we use chemical data from anoxic Limfjorden and Peru Margin sediments (*9*, *70*). To address the uncertainty in our results arising from spatial variability of these conditions, we ran additional simulations, where the concentrations of the reactants and products were varied to represent high-energy and low-energy scenarios (see Supplementary Text).

### Cell abundance

Global subseafloor cell abundance was estimated on the basis of a power law formulation from (*1*)

$$Y=b\cdot z^{m}$$ (31)

where *Y* (cells per cubic centimeter) is cell abundance, *z* (meters) denotes the depth below the seafloor, and *b* and *m* are parameters based on mean sedimentation rate and distance from landmasses greater than 10^5^ km^2^. *b* and *m* were interpolated onto our 0.25° × 0.25° global grid based on data provided in (*1*). Cell concentration in the uppermost bioturbated layer (*Y*_*z*bio_, 0 to 10 cm) was uniformly described, following (*1*), as

$$Y_{z\text{bio}}=b\cdot 0.1^{m}$$ (32)

### Global totals

We quantified total subseafloor sedimentary volume, POC degradation, and cell abundance by integrating across specific sediment depth and age horizons and by summing across the global grid representing the entire ocean floor.

## Supplementary Material

aba0697_SM.pdf

## SUPPLEMENTARY MATERIALS

Supplementary material for this article is available at http://advances.sciencemag.org/cgi/content/full/6/32/eaba0697/DC1

## Appendix

View/request a protocol for this paper from *Bio-protocol*.
