# Supplementary material for: Widespread energy limitation to life in global subseafloor sediments
Source: Sci Adv. 2020 Aug 5;6(32):eaba0697. doi: 10.1126/sciadv.aba0697 (PMC7406382; doi:10.1126/sciadv.aba0697)
Supplement: aba0697_SM.pdf [file aba0697_SM.pdf]

Science Advances
AAAS

advances.sciencemag.org/cgi/content/full/6/32/eaba0697/DC1

Supplementary Materials for

Widespread energy limitation to life in global subseafloor sediments

J. A. Bradley*, S. Arndt, J. P. Amend, E. Burwicz, A. W. Dale, M. Egger, D. E. LaRowe

*Corresponding author. Email: jbradley.earth@gmail.com

Published 5 August 2020, *Sci. Adv.* **6**, eaba0697 (2020)
DOI: 10.1126/sciadv.aba0697

This PDF file includes:

Supplementary Text
Figs. S1 to S5
Tables S1 to S4
References

Supplementary Text

Partitioning of the ocean floor

We partition the ocean floor into shelf, margin and abyss domains (Fig. S5). The locations of the continental margin boundaries are adopted from ref. (*71*): shelf environments roughly correspond to water depths <200m, with the exception of the Antarctic region where shelf area corresponds to water depths <500m; areas deeper than ~3500m are taken to be abyssal plain; ocean floor covered by 500–3500m water are referred to as margins. Accordingly, continental shelf underlies about 6.3% of ocean surface area, margins make up 10.8% and the abyssal domain constitutes the remaining 82.9%.

Power produced by global marine sediment microbes

In order to calculate the total power produced in the (i) oxic, (ii) sulfate-reducing and (iii) methanogenic sedimentary layers, first we compute the total integrated rate of POC degradation between (i) the SWI and the O_2-penetration depth, (ii) the O_2-penetration depth and the sulfate-methane transition depth, and (iii) the sulfate-methane transition depth and the maximum depth of quaternary sediments. This is then multiplied by the Gibbs energy of each catabolic reaction (Table S2). Total power produced by microorganisms in Quaternary age marine sediment is 37.3 GW. Large uncertainties associated with the POC burial flux prior to 2.59 million years ago obscures efforts to model degradation rates in pre-Quaternary sediments. Thus, the overall contribution of power from microbially mediated POC globally remains unknown. However, Quaternary sediments include the most microbially active portions of marine sediments, producing orders of magnitude higher power than sediments older than 2.59 million years. Thus, it is expected that sediments deposited before the Pleistocene produce negligible amounts of power (Fig. S2) compared to Pleistocene-age sediments, and thus our value stands as a first-order estimate for the total power of all marine-sediment hosted life. Assuming the total power captured by photosynthesis is 100 TW

(*22*), and that net primary productivity in the ocean accounts for 46.2% of global total (*23*), marine sediment microorganisms utilize the equivalent of ~0.08% of all photosynthetically produced power in the marine photic zone.

Uncertainty

We use a combination of modelling approaches to derive estimates of cell-specific energy turnover on a global scale. Under nominal conditions and assumptions, we find that microorganisms subsist at a median of 3.3×10^{-20} W $cell^{-1}$, and 84.0% of microorganisms subsist at an energy flux less than 1.9×10^{-19} W $cell^{-1}$. Uncertainties in our calculations arise from a number of factors that are addressed here. Calculated cell-specific power production depends on (i) the rate of POC degradation, (ii) the Gibbs energy of the reaction, and (iii) the number of cells carrying out the reaction. Uncertainty in each of these factors, tested individually, results in a range in median power per cell from 1.42×10^{-20} W to 6.82×10^{-20} W. The percentage of microorganisms subsisting at a power below 1.9×10^{-19} W $cell^{-1}$ is at least 70.4% in all simulations. In addition, we have quantified the possible propagation of uncertainty through each component of our model. We find when accounting for the propagation of uncertainty (i-iii) under the most cautious/prudent assumptions (i.e. the combination of parameters that provide the highest deviation from nominal results), median per cell power is 1.42×10^{-20} to 10.20×10^{-20} W. Considering uncertainty in all factors, our main conclusions are supported, given the extraordinarily low per-cell powers, as well as relatively minor deviation from nominal results. The specific factors (i-iii) and analyses are outlined below.

(i) POC degradation rate.

In order to address the uncertainty associated with modelled POC degradation rates, we evaluate a range of simulations encapsulating all possible variability in the reactivity of POC deposited onto the seafloor and its evolution during burial. These phenomena are accounted for within the RCM by two parameters: a representing the average lifetime of reactive components of the POC, and v, a dimensionless parameter determining the shape of the distribution of reactivities. These adjustable parameters completely determine the shape of the initial distribution of POC reactivity. High v and low a values indicate a mixture of POC dominated by compounds that are typically degraded rapidly. Low v and high a values indicate a larger fraction of less reactive compounds that degrade slowly. See ref. (*29*) for a more in-depth description of these parameters. We prescribe POC reactivity for each domain, constrained based on a global parameter compilation, and observations that the v parameter values do not vary much between sites, while the a parameter can vary over orders of magnitude (e.g. refs. (*29,41*)). Therefore, we designate a constant v parameter of 0.125, characteristic of fresh organic matter (*29*) for all three domains. The values of a are determined by sedimentation rates based on a global compilation (*29*):

$$a = 10^{(3.35-14.81\cdot\omega)} \tag{33}$$

where ω represents the sedimentation rate (cm yr^{-1}). This approach accounts for order-of-magnitude changes in a due to factors that control organic matter transit times from its source to deposition. These parameters thus reflect typically observed RCM parameter variability across various depositional environments. Despite our approach relying on a well-established model with hundreds of existing applications that are validated using a large body of existing interdisciplinary data, the availability of more coordinated data from a range of contrasting environments will serve to advance our predictive capabilities further in the future.

We also run simulations using parameter values spanning nearly the entire range of observed values reported in the literature (e.g. refs. (*29,41*)) (Table S3). After evaluating all possible variability in the reactivity of POC deposited onto the seafloor and its evolution during burial, the median power per cell ranges from 1.42×10^{-20} W (high reactivity scenario) to 4.02×10^{-20} W (low reactivity scenario). The percentage of microbes subsisting at powers $<1.9\times10^{-19}$ W $cell^{-1}$ is 77.5% (under a high reactivity scenario) and 84.6% (under a low reactivity scenario), representing a deviation from nominal simulation results by 0.6% to -6.5%. The values of a used in our uncertainty analyses extend well past what would be calculated based on the sedimentation-rate relationship (Eq. 33). The large parameter range that is tested has only a minor bearing on per cell energy fluxes.

(ii) Gibbs energy of the reaction.
In order to address the uncertainty associated with variability in Gibbs energy arising from spatial heterogeneities in the concentrations of reactants and products, we run additional simulations where the concentrations of the reactants and products are varied over realistic ranges to represent high energy and low energy scenarios (Table S3). The uncertainty in the Gibbs energies of each reaction does not meaningfully affect our results, considering microbial power production spans a range of five orders of magnitude, and the deviation of Gibbs energies from our nominal values is <20% and in many cases <1%. Accounting for the widest range of uncertainty in Gibbs energy, the median power per cell spans a range from 2.61×10^{-20} W (low energy scenario) to 4.26×10^{-20} W (high energy scenario). The percentage of microbes subsisting at powers $<1.9\times10^{-19}$ W $cell^{-1}$ is 81.7% to 87.2%, or a deviation from nominal simulation results by only 3.2% to -2.4%. Therefore, the reflected range in possible Gibbs energies and power do not meaningfully affect our results.

(iii) Number of cells carrying out the reaction.
We use the best available spatially-resolved global model for subseafloor biomass distribution (*1*) to estimate cell abundance and thus calculate per-cell rates. We designate POC degraded in oxic sediments to be mediated by O_2, POC degraded in sulfate-reducing sediments (deeper than the maximum O_2 penetration depth but shallower than the sulfate-methane transition) to be mediated by SO_4^{2-}, and prescribe POC degraded beneath the sulfate-methane transition to methanogenesis (Table S2). Thus, the reaction network is simplified to account for the most pertinent biogeochemical process (*53*) and the major catabolic pathways of POC oxidation (*54*). Cell-specific catabolic rates are calculated by dividing total power by total cell numbers, thus assuming that all cells are equally performing the catabolic metabolism associated with that particular sedimentary zone (i.e. aerobic heterotrophy in the oxic zone, sulfate reduction in the sulfate-reducing zone, methanogenesis in the methanogenic zone, as in Table S2).

A large body of evidence suggests that the vast majority of microorganisms in subsurface sediments are involved in the degradation of organic matter (and overwhelmingly hold the genetic potential to degrade organic molecules), especially in organic-rich, coastal and/or anoxic settings (*4,26,72-80*). Oxic sediments are known to contain microbial communities consisting of strictly aerobic and facultative anaerobic heterotrophs, whilst anoxic sediments are enriched with strictly anaerobic groups, such as sulfate-reducing bacteria, anaerobic members of the Chloroflexi phylum, and methanogenic and methanotrophic archaea (*78*). We designate that POC degradation in the methanogenic sediment layers is carried out by acetoclastic methanogenesis, whilst noting that hydrogenotrophic methanogenesis has been shown to occur deep in the seafloor (*12*). Beulig et al. (*26*) have recently shown that hydrogenotrophic methanogens are ultimately fueled by acetate: acetate is converted to CO_2

and H_2, which is then used by methanogens, and thus, our assumptions serve the present study well. Besides the major POC degradation pathways used here (aerobic heterotrophy, sulfate reduction, and methanogenesis), denitrification, metal oxide reduction, weathering reactions, seismic processes, radioactive decay, and secondary redox reactions associated with POC degradation may all directly or indirectly (i.e. produce reactants that when utilized by microorganisms) supply energy (*28*). Energy may also be provided by fermentation, although its importance is not well known. Although POC oxidation is the primary energy supply to microorganisms, we run additional simulations where we assume that only 50% to 95% of the microbial community are responsible for and therefore gaining energy from POC degradation (*81-83*). Accurate determination of the proportion of organisms that are heterotrophic in a marine sediment sample is plagued with various problems including difficulty to assign function based on genomic reads, variability among and between genomic datasets, the varying number of marker genes for heterotrophy relative to carbon fixation (within single cells and among different organisms), the number of pathways involved in organic matter degradation in sediments, and the difficulty associated with normalization to single copy marker genes (*84-89*). Moreover, many of the phylogenetic groups found in sediments are candidate phyla that are not yet cultivated or closely related to a pure culture (*12,55,79,90*). These factors make determination of metabolic function challenging even for a single sample of marine sediment – let alone doing this on a global basis. For these reasons, we are cautious about suggesting precise values for the proportion of the microbial community are responsible for and therefore gaining energy from POC degradation on a global scale. Instead, we evaluate a wide range of uncertainty in the proportion of organisms assumed to be utilizing organic carbon for energy (95% to 50%). Based on widely-accepted views of marine sediment biogeochemistry and the data provided above, we are confident that our analysis of uncertainty spans a sufficiently wide range. Moreover, selecting a lower-bound global estimate of 50% has an additional advantage of clearly demonstrating the consequence of halving the number of cells being considered in our model. After evaluating possible variability in the proportion of heterotrophic microorganisms in subseafloor sediments, the median power per cell is 3.49×10^{-20} W (95% heterotrophs) to 6.64×10^{-20} W (50% heterotrophs). Furthermore, the percentage of microbes subsisting at powers $<1.9\times10^{-19}$ W $cell^{-1}$ is 70.4% (assuming 50% heterotrophs) to 83.3% (assuming 95% heterotrophs), representing a maximum deviation from nominal simulation results by -13.6%. It is noteworthy that implementing this extreme end-member limit causes only a minor difference in our results, and nevertheless supports our conclusion that energy limitation to microbial life is extremely widespread in marine sediments. Moreover, if we have underestimated the number of cells contained in marine sediment (based on ref. (*1*)), per cell power utilization will be even lower – thereby strengthening our overall conclusion of widespread energy limitation to life in marine sediments.

We also run additional simulations to evaluate how changes in the geographic location and depth of occurrence of each metabolic zone (i.e. oxygen penetration depth, sulfate-methane transition depth) affect our results. With 10% variation in the depth of oxic, sulfate-reducing, and methanogenic sediments, the impact on the median per cell power utilization is trivial: a change from 3.41×10^{-20} W to 3.42×10^{-20}.

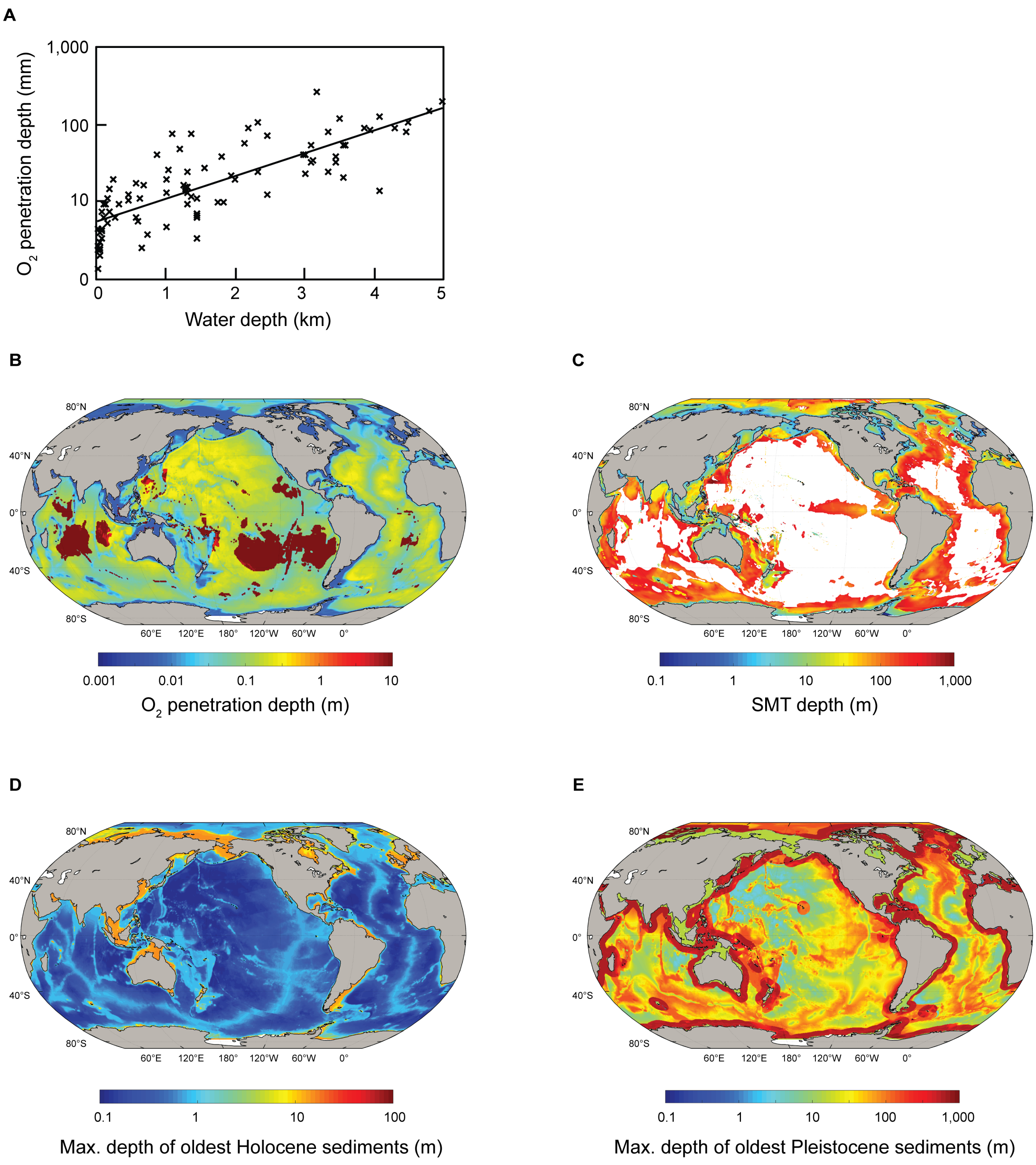

Fig S1. Oxygen penetration, sulfate-methane transition, and extent of Holocene and Pleistocene-aged sediments. (A) Regression model relating measured O_2 penetration depth in global sediments (y) to water depth (x): $y = 5.5673 \times e^{0.6769x}$ (R^2=0.63). Supporting data and references are provided in Table S4. These data were compiled by Ronnie Glud (University of Southern Denmark) and Frank Wenzhöfer (Alfred Wegener Institute, Bremerhaven). (B) Modelled oxygen penetration depth (m) in global sediments. (C) Sulfate-methane transition depth (m) in global sediments. White areas denote regions of the seafloor where there is no sulfate-methane transition. (D) Maximum depth (m) of the oldest Holocene-aged and (E) Pleistocene-aged sediments.

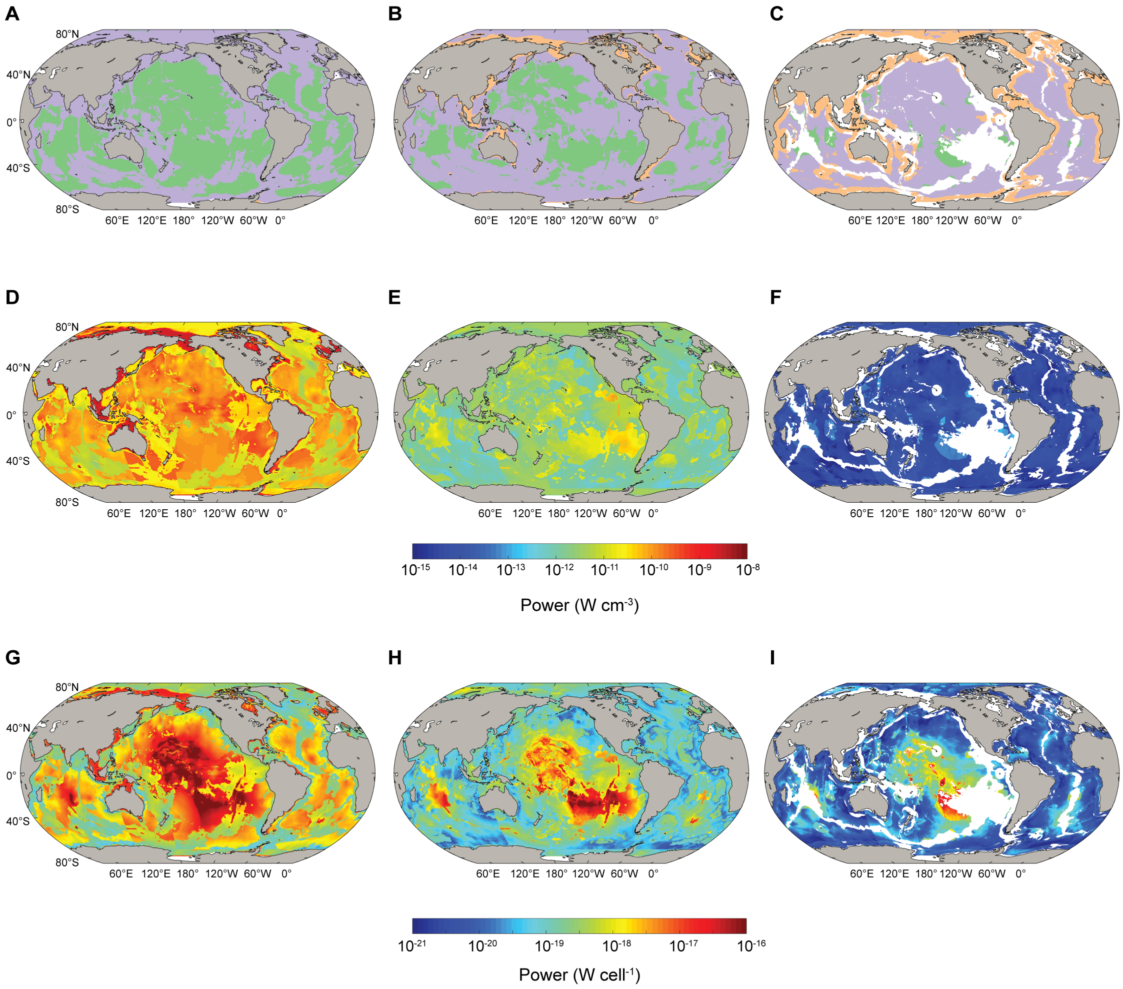

Fig. S2. Global distribution of catabolic zones and microbial power production. Global maps depicting (A-C) the major catabolic pathway of OC oxidation at 10cm beneath the seafloor, the layer of sediment deposited at the beginning of the Holocene (11,700 years ago), and the layer of sediment deposited at the beginning of the Pleistocene (2.59 million years ago). Green shading represents oxic sediments, lilac denotes sulfate-reducing sediments, and orange signifies methanogenic sediments; (D-F) Power produced by microbial POC degradation (W cm^{-3}) in sediment 10 cm beneath the seafloor, 11,700 year-old sediment, and 2.59 million year-old sediment respectively. (G-I) Microbial power production (W $cell^{-1}$) in sediment 10 cm beneath the seafloor, 11,700 year-old sediment, and 2.59 million year-old sediment respectively. White shading (C, F, I) represents locations where there is no Pleistocene sediment (i.e. sediment age does not extend to (or beyond) the beginning of the Pleistocene).

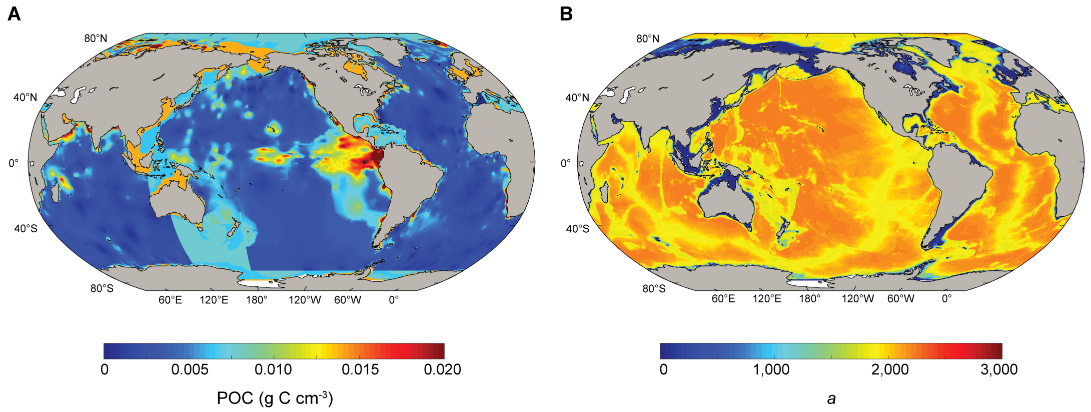

Fig. S3. Geographical distribution and reactivity of POC at the sediment-water interface. Global maps depicting (A) the POC concentration at the SWI (g C cm^{-3}) and (B) the reactive continuum model parameter *a* (years), representing the average lifetime of reactive components of the POC. Low *a* values indicate a mixture of POC dominated by more recently formed (i.e. younger) compounds that are typically degraded rapidly, whereas high *a* values indicate a larger fraction of ancient compounds that degrade slowly.

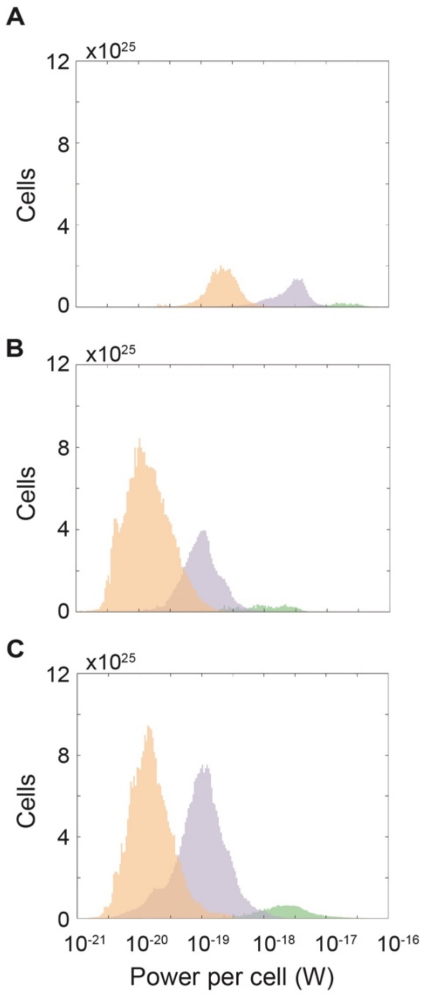

Fig S4. Frequency distribution of microbial power production. Microbial power production in (A) continental shelf sediments, (B) margins sediments and (C) abyssal sediments. Green denotes oxic sediment, lilac signifies sulfate-reducing sediment, and orange represents methanogenic sediment.

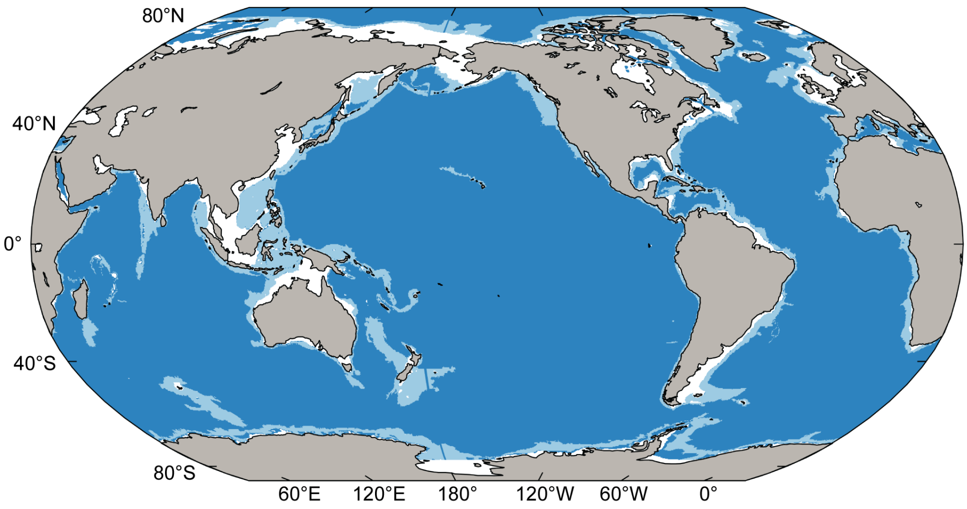

Fig S5. Geographic distribution of major sediment domains. Shelf (white), margin (light blue) and abyss (dark blue) domains considered in this study.

Reaction no.	Metabolism	Reaction
1	Aerobic heterotrophy	$C_2H_3O_2^- + 2O_2 \rightleftharpoons H^+ + 2HCO_3^-$
2	Sulfate reduction	$C_2H_3O_2^- + SO_4^{2-} \rightleftharpoons HS^- + 2HCO_3^-$
3	Methanogenesis	$C_2H_3O_2^- + H_2O \rightleftharpoons CH_4 + HCO_3^-$

Table S1. Catabolic pathways for POC oxidation considered in this study.

Metabolism	ΔG_r	$C_2H_3O_2^-$	O_2	HCO_3^-	SO_4^{2-}	HS^-	CH_4	pH
Aerobic heterotrophy								
Nominal	-34.7200	0.00063[a,b]	0.00008[c]	0.0023[c]				7.78[c]
Low energy	-34.4630	0.00020[a,b]	0.00005[c]	0.0020[c]				7.78[c]
High energy	-34.8129	0.00100[a,b]	0.00011[c]	0.0026[c]				7.78[c]
Sulfate reduction								
Nominal	-2.6833	0.00063[a,b]		0.0250[d]	0.0154[e]	1×10^{-6} [d]		
Low energy	-2.1370	0.00020[a,b]		0.0400[d]	0.0028[e]	5×10^{-6} [d]		
High energy	-2.9842	0.00100[a,b]		0.0100[d]	0.0280[e]	1×10^{-7} [d]		
Methanogenesis								
Nominal	-0.9872	0.00063[a,b]		0.0280[d]			0.00060[d]	
Low energy	-0.7735	0.00020[a,b]		0.0330[d]			0.00100[d]	
High energy	-1.3406	0.00100[a,b]		0.0230[d]			0.00002[d]	

Table S2. Gibbs energies and concentrations of reactants and products used to calculate the Gibbs energies for each catabolic reaction, as well as to address uncertainty in these values. Gibbs energies are provided in kJ g^{-1} C. Concentrations are provided in moles (M). Concentrations for the various scenarios are taken from the literature and are intended to represent the variation that is present in typical sedimentary settings and not extreme values that are not globally representative. [a] ref. (*91*); [b] Ref. (*92*); [c] Ref. (*2*); [d] Ref. (*70*); [e] Ref. (*9*).

Parameter	Definition	Shelf	Margin	Abyss	Units
Φ_0	Sediment porosity at the sediment-water interface[a]	0.45 [a]	0.74 [a]	0.70 [a]	
c_0	Sediment compaction length scale [b]	0.50×10^{-3} [b]	1.70×10^{-4} [b,c]	0.85×10^{-3} [b]	m^{-1}
a	Reactive continuum age parameter				years
	Nominal	$10^{(3.35-14.81\cdot\omega)}$ [d]	$10^{(3.35-14.81\cdot\omega)}$ [d]	$10^{(3.35-14.81\cdot\omega)}$ [d]	
	Low reactivity	5.0 [e]	3.0×10^{3} [f]	3.5×10^{4} [g]	
	High reactivity	3.0×10^{-4} [h]	3.0×10^{-4} [h]	20.0	
v	Reactive continuum distribution parameter				
	Nominal	0.125	0.125	0.125	
	Low reactivity	0.135 [e]	0.160 [f]	0.160 [g]	
	High reactivity	0.125 [h]	0.125 [h]	0.160 [i]	

Table S3. Parameter values used to characterize the porosity and organic matter content of continental shelf, margin and abyss domains of marine sediments.
[a] Ref. (93); [b] These values are representative of a sandstone-siltstone mixture (shelf), a sandstone-siltstone- shale combination (margin) and typical shales and biogenic-dominated sediments (abyss) (*49*); [c] Ref. (*37*); [d] based on global compilation by ref. (*28*), *ω* represents sedimentation rate (cm yr^{-1}); [e] Ref. (*94*), Arkona Basin; [f] Ref. (*95*), Sea of Okhotsk; [g] Ref. (*96*), Central Pacific; [h] Refs. (*41,97*), fresh plankton material from Long Island Sound; [i] Ref. (*98*), Peru.

Authors & Year	Journal	Reference	Station	Water Depth (km)	Oxygen Penetration Depth (mm)	Lat	Long
Glud et al, 1994	DSR I 41, 1767-1788	(*99*)	1702	3.107	33.0	-6.5533	10.3167
Glud et al, 1994	DSR I 41, 1767-1788	(*99*)	1703	1.747	10.0	-17.4467	11.0133
Glud et al, 1994	DSR I 41, 1767-1788	(*99*)	1708	2.321	25.0	-20.0967	8.9667
Glud et al, 1994	DSR I 41, 1767-1788	(*99*)	1711	1.947	22.0	-23.3400	12.3817
Glud et al, 1994	DSR I 41, 1767-1788	(*99*)	1713	0.604	5.6	-23.2383	13.0133
Glud et al, 1994	DSR I 41, 1767-1788	(*99*)	1719	1.015	13.0	-28.9517	14.1750
Glud et al, 1994	DSR I 41, 1767-1788	(*99*)	1721	3.095	55.0	-29.1933	13.0900
Glud et al, 1994	DSR I 41, 1767-1788	(*99*)	1723	4.986	200.0	-29.8850	8.6650
Glud et al, 1999	MEPS 186: 9-20	(*51*)	GeoB 3323	3.459	37.8	-43.2167	-76.0000
Glud et al, 1999	MEPS 186: 9-22	(*51*)	GeoB 3337	4.079	129.7	-35.2500	-86.0000
Glud et al, 1999	MEPS 186: 9-23	(*51*)	GeoB 3350	2.470	12.6	-35.2550	-73.4167
Glud et al, 1999	MEPS 186: 9-24	(*51*)	GeoB 3355	1.445	11.1	-35.2167	-73.1117
Glud et al, 1999	MEPS 186: 9-25	(*51*)	GeoB 3356	0.580	6.5	-35.2183	-73.2033
Glud et al, 2003	LaO 48(3): 1265-1276	(*100*)	6	0.016	2.5	56.1517	10.3200
Glud et al, 2003	LaO 48(3): 1265-1276	(*100*)	6	0.016	4.5	56.1517	10.3200
Glud et al, 1998	MEPS 173: 237-251	(101)	SV-1	0.329	9.9	69.4900	-18.1250
Glud et al, 1998	MEPS 173: 237-252	(*101*)	SV-2	0.155	5.5	76.9700	-15.5750
Glud et al, 1998	MEPS 173: 237-253	(*101*)	SV-3	0.115	9.6	77.7633	-15.0650
Glud et al, 1998	MEPS 173: 237-254	(*101*)	SV-4	0.138	9.3	76.9617	-15.0033
Glud et al, 1998	MEPS 173: 237-255	(*101*)	SV-5	0.175	10.9	77.5500	-19.0833
Wenzhoefer et al, 2001a	*DSR I 48: 1741-1755*	(*102*)	4401	3.350	80.0	4.7600	-43.7583
Wenzhoefer et al, 2001a	*DSR I 48: 1741-1755*	(*102*)	4409	3.850	90.0	3.6417	-45.2400
Wenzhoefer et al, 2001a	*DSR I 48: 1741-1755*	(*102*)	4417	3.510	120.0	5.1450	-46.5600
Wenzhoefer et al, 2001a	*DSR I 48: 1741-1755*	(*102*)	4419	4.486	110.0	9.6700	-54.2583
Wenzhoefer et al, 2001a	*DSR I 48: 1741-1755*	(102)	4421	3.180	260.0	16.9850	-46.0150
Wenzhoefer et al, 2001b	GCA 65(16): 2677-2690	(*103*)	4909	1.310	25.0	-2.0733	8.6267
Wenzhoefer et al, 2001b	GCA 65(16): 2677-2690	(*103*)	4913	1.300	15.0	-5.5000	11.0733
Wenzhoefer et al, 2001b	GCA 65(16): 2677-2690	(*103*)	4917	1.300	14.0	-11.9067	13.0717
Wenzhoefer et al, 2001b	GCA 65(16): 2677-2690	(*103*)	6214	1.566	28.0	-34.5200	-51.4383
Wenzhoefer et al, 2001b	GCA 65(16): 2677-2690	(*103*)	6219	3.550	55.5	-35.1850	-50.5650
Wenzhoefer et al, 2001b	GCA 65(16): 2677-2690	(*103*)	6229	3.442	33.5	-37.2133	-52.6517

Wenzhoefer et al, 2001b	GCA 65(16): 2677-2690	(*103*)	4901	2.185	90.0	2.6800	6.7300
Wenzhoefer et al, 2001b	GCA 65(16): 2677-2690	(*103*)	4906	1.275	15.5	-0.6867	8.3817
Jahnke et al, 1989	GCA 53: 2947-2960	(*104*)	J4	1.007	20.0	9.2383	-16.9950
Jahnke et al, 1989	GCA 53: 2947-2960	(*104*)	J6	4.470	80.0	8.3833	-17.6100
Wenzhöfer & Glud, 2002	DSR I 49: 1255-1279	(*105*)	2101	1.809	38.0	-23.9950	-41.2083
Wenzhöfer & Glud, 2002	DSR I 49: 1255-1279	(*105*)	2103	1.047	26.0	-27.1750	-46.4633
Wenzhöfer & Glud, 2002	DSR I 49: 1255-1279	(*105*)	3705	1.305	13.0	-24.3117	12.9983
Wenzhöfer & Glud, 2002	DSR I 49: 1255-1279	(*105*)	3706	1.323	16.0	-22.7250	12.6050
Wenzhöfer & Glud, 2002	DSR I 49: 1255-1279	(*105*)	3707	1.355	11.6	-21.6300	12.1967
Wenzhöfer & Glud, 2002	DSR I 49: 1255-1279	(*105*)	3718	1.310	9.7	-24.8983	13.1667
Wenzhöfer & Glud, 2002	DSR I 49: 1255-1279	(*105*)	3719	1.995	19.8	-24.9983	12.8667
Wenzhöfer & Glud, 2002	DSR I 49: 1255-1279	(*105*)	3721	3.017	22.8	-28.1467	12.4033
Wenzhöfer & Glud, 2002	DSR I 49: 1255-1279	(*105*)	4234	1.362	75.0	28.8883	-13.2250
Witte et al, 2003	MEPS 251: 27-36	(*106*)	Sognefjor d	1.265	16.8	61.1420	6.0203
Witte et al, 2003	Nature 424, 763-766	(*107*)	PAP	4.800	150.0	48.8333	-16.5833
Black et al, 2001	Cont. Shelf Res. 21, 859-877	(*108*)	A	1.100	78.0	52.9068	-16.9095
Black et al, 2001	Cont. Shelf Res. 21, 859-877	(*108*)	B	3.570	21.0	57.3885	-15.7107
Glud et al, 2009	LaO 54 (1), 1-12	(*109*)	OBBII 09/03	1.450	3.5	35.0143	139.3598
Glud et al, 2009	LaO 54 (1), 1-12	(*109*)	OBBII 03/06	1.450	6.3	35.0143	139.3598
Glud et al, 2009	LaO 54 (1), 1-12	(*109*)	OBBII 12/06	1.450	6.6	35.0143	139.3598
Glud et al, 2009	LaO 54 (1), 1-12	(*109*)	OBBII 01/08	1.450	7.1	35.0143	139.3598
Giordani et al, 2002	J of Marine Systems 33-23: 365-387	(*110*)	P1	0.246	20.0	42.8500	14.7500
Giordani et al, 2002	J of Marine Systems 33-23: 365-387	(*110*)	A1	1.196	50.0	41.8457	17.7452
Giordani et al, 2002	J of Marine Systems 33-23: 365-387	(*110*)	O2	0.870	40.0	39.8287	18.9580
Lansard et al, 2008	Cont. Shelf Res. 28, 1501-1510	(*107*)	Rhone-1	0.063	2.6	43.3067	4.8850
Lansard et al, 2008	Cont. Shelf Res. 28, 1501-1510	(*111*)	Rhone-2	0.098	6.4	43.2417	4.8972
Lansard et al, 2008	Cont. Shelf Res. 28, 1501-1510	(*111*)	Sofi	0.192	14.5	43.0667	5.1267
Lansard et al, 2009	ECSS 81, 544-554	(*112*)	1	0.020	1.4	43.3150	4.8533
Lansard et al, 2009	ECSS 81, 544-554	(*112*)	3	0.053	2.4	43.3050	4.8350

Lansard et al, 2009	ECSS 81, 544-554	(*112*)	4	0.060	3.0	43.3033	4.8583
Lansard et al, 2009	ECSS 81, 544-554	(*112*)	5	0.063	2.1	43.3067	4.8850
Lansard et al, 2009	ECSS 81, 544-554	(*112*)	6	0.040	2.8	43.2950	4.7500
Lansard et al, 2009	ECSS 81, 544-554	(*112*)	13	0.075	4.5	43.2717	4.7750
Lansard et al, 2009	ECSS 81, 544-554	(*112*)	14	0.085	3.4	43.2650	4.8217
Lansard et al, 2009	ECSS 81, 544-554	(*112*)	16	0.083	7.4	43.2683	4.9683
Lansard et al, 2009	ECSS 81, 544-554	(*112*)	17	0.073	4.6	43.2483	4.7317
Lansard et al, 2009	ECSS 81, 544-554	(*112*)	22	0.074	4.2	43.2200	4.6967
Sachs et al, 2009	DSR I 56, 1319-1335	(*113*)	85-1	2.995	42.0	-52.0190	0.0012
Sachs et al, 2009	DSR I 56, 1319-1335	(*113*)	84-1	3.004	42.0	-52.2003	0.0028
Sachs et al, 2009	DSR I 56, 1319-1335	(*113*)	47-1	1.843	10.0	-69.6733	1.0373
Sachs et al, 2009	DSR I 56, 1319-1335	(*113*)	39-4	2.125	57.0	-64.5032	2.8760
Sachs et al, 2009	DSR I 56, 1319-1335	(*113*)	705-1	4.293	90.0	-49.0017	12.2550
Sachs et al, 2009	DSR I 56, 1319-1335	(*113*)	703-1	3.330	25.0	-52.5850	9.0033
Sachs et al, 2009	DSR I 56, 1319-1335	(*113*)	600-1	3.589	55.0	-49.9983	2.3367
Sachs et al, 2009	DSR I 56, 1319-1335	(*113*)	598-1	3.942	85.0	-49.3083	2.1950
Sachs et al, 2009	DSR I 56, 1319-1335	(*113*)	972	4.091	14.0	-48.5017	-6.0033
Sachs et al, 2009	DSR I 56, 1319-1335	(*113*)	891	3.117	35.0	-55.0083	-6.0050
Canfield et al, 1993	Mar Geol 113 27-40	(*114*)	S4	0.190	7.5	58.1695	10.0727
Canfield et al, 1993	Mar Geol 113 27-40	(*114*)	S9	0.695	17.0	58.6227	10.2878
Larsen et al,	Unpublished			0.650	2.6	-12.5125	-77.5695
Larsen et al,	Unpublished			0.750	3.9	-12.5238	-77.5827
Larsen et al,	Unpublished			1.000	4.7	-12.5897	-77.6832
Wenzhoefer et al,	Unpublished			2.470	74.0	79.0070	4.3317
Wenzhoefer et al,	Unpublished			2.340	109.0	79.0667	4.0018
Glud et al.	Unpublished		St 13	0.630	11.4	64.3667	-51.6233
Glud et al.	Unpublished		St 17	0.579	17.6	64.6100	-50.9583
Glud et al.	Unpublished		St 20	0.476	10.8	64.6800	-50.2883
Glud et al.	Unpublished		St 14	0.453	12.4	64.4200	-51.5100
Glud et al.	Unpublished		St 8	0.273	6.2	63.9500	-52.3667
Glud et al.	Unpublished		LE	0.060	4.0	59.4555	5.1875

Table S4. Supporting data used to inform regression model of O_2 penetration depth in global sediments. These data were compiled by Ronnie Glud (University of Southern Denmark) and Frank Wenzhöfer (Alfred Wegener Institute, Bremerhaven).

REFERENCES AND NOTES

1. J. Kallmeyer, R. Pockalny, R. R. Adhikari, D. C. Smith, S. D'Hondt, Global distribution of microbial abundance and biomass in subseafloor sediment. *Proc. Natl. Acad. Sci. U.S.A.* **109**, 16213–16216 (2012).

2. S. D'Hondt, F. Inagaki, C. A. Zarikian, L. J. Abrams, N. Dubois, T. Engelhardt, H. Evans, T. Ferdelman, B. Gribsholt, R. N. Harris, B. W. Hoppie, J.-H. Hyun, J. Kallmeyer, J. Kim, J. E. Lynch, C. C. McKinley, S. Mitsunobu, Y. Morono, R. W. Murray, R. Pockalny, J. Sauvage, T. Shimono, F. Shiraishi, D. C. Smith, C. E. Smith-Duque, A. J. Spivack, B. O. Steinsbu, Y. Suzuki, M. Szpak, L. Toffin, G. Uramoto, Y. T. Yamaguchi, G.-l. Zhang, X.-H. Zhang, W. Ziebis, Presence of oxygen and aerobic communities from sea floor to basement in deep-sea sediments. *Nat. Geosci.* **8**, 299–304 (2015).

3. B. B. Jørgensen, I. P. G. Marshall, Slow microbial life in the seabed. *Ann. Rev. Mar. Sci.* **8**, 311–332 (2016).

4. K. G. Lloyd, L. Schreiber, D. G. Petersen, K. U. Kjeldsen, M. A. Lever, A. D. Steen, R. Stepanauskas, M. Richter, S. Kleindienst, S. Lenk, A. Schramm, B. B. Jørgensen, Predominant archaea in marine sediments degrade detrital proteins. *Nature* **496**, 215–218 (2013).

5. J. S. Lipp, Y. Morono, F. Inagaki, K.-U. Hinrichs, Significant contribution of Archaea to extant biomass in marine subsurface sediments. *Nature* **454**, 991–994 (2008).

6. H. Røy, J. Kallmeyer, R. R. Adhikari, R. Pockalny, B. B. Jørgensen, S. D'Hondt, Aerobic microbial respiration in 86-million-year-old deep-sea red clay. *Science* **336**, 922–925 (2012).

7. R. J. Parkes, B. Cragg, E. Roussel, G. Webster, A. Weightman, H. Sass, A review of prokaryotic populations and processes in sub-seafloor sediments, including biosphere:geosphere interactions. *Mar. Geol.* **352**, 409–425 (2014).

8. D. H. Rothman, Atmospheric carbon dioxide levels for the last 500 million years. *Proc. Natl. Acad. Sci. U.S.A.* **99**, 4167–4171 (2002).

9. S. D’Hondt, B. B. Jørgensen, D. J. Miller, A. Batzke, R. Blake, B. A. Cragg, H. Cypionka, G. R. Dickens, T. Ferdelman, K.-U. Hinrichs, N. G. Holm, R. Mitterer, A. Spivack, G. Wang, B. Bekins, B. Engelen, K. Ford, G. Gettemy, S. D. Rutherford, H. Sass, C. G. Skilbeck, I. W. Aiello, G. Guèrin, C. H. House, F. Inagaki, P. Meister, T. Naehr, S. Niitsuma, R. J. Parkes, A. Schippers, D. C. Smith, A. Teske, J. Wiegel, C. N. Padilla, J. L. S. Acosta, Distributions of microbial activities in deep subseafloor sediments. *Science* **306**, 2216–2221 (2004).

10. B. B. Jørgensen, S. D’Hondt, A starving majority deep beneath the seafloor. *Science* **314**, 932–934 (2006).

11. E. Trembath-Reichert, Y. Morono, A. Ijiri, T. Hoshino, K. S. Dawson, F. Inagaki, V. J. Orphan, Methyl-compound use and slow growth characterize microbial life in 2-km-deep subseafloor coal and shale beds. *Proc. Natl. Acad. Sci. U.S.A.* **114**, E9206–E9215 (2017).

12. F. Inagaki, K.-U. Hinrichs, Y. Kubo, M. W. Bowles, V. B. Heuer, W.-L. Hong, T. Hoshino, A. Ijiri, H. Imachi, M. Ito, M. Kaneko, M. A. Lever, Y.-S. Lin, B. A. Methé, S. Morita, Y. Morono, W. Tanikawa, M. Bihan, S. A. Bowden, M. Elvert, C. Glombitza, D. Gross, G. J. Harrington, T. Hori, K. Li, D. Limmer, C.-H. Liu, M. Murayama, N. Ohkouchi, S. Ono, Y.-S. Park, S. C. Phillips, X. Prieto-Mollar, M. Purkey, N. Riedinger, Y. Sanada, J. Sauvage, G. Snyder, R. Susilawati, Y. Takano, E. Tasumi, T. Terada, H. Tomaru, E. Trembath-Reichert, D. T. Wang, Y. Yamada, Exploring deep microbial life in coal-bearing sediment down to ~2.5 km below the ocean floor. *Science* **349**, 420–424 (2015).

13. D. E. LaRowe, J. P. Amend, Power limits for microbial life. *Front. Microbiol.* **6**, 718 (2015).

14. Y. Morono, T. Terada, M. Nishizawa, M. Ito, F. Hillion, N. Takahata, Y. Sano, F. Inagaki, Carbon and nitrogen assimilation in deep subseafloor microbial cells. *Proc. Natl. Acad. Sci. U.S.A.* **108**, 18295–18300 (2011).

15. P. Starnawski, T. Bataillon, T. J. G. Ettema, L. M. Jochum, L. Schreiber, X. Chen, M. A. Lever, M. F. Polz, B. B. Jørgensen, A. Schramm, K. U. Kjeldsen, Microbial community assembly and evolution in subseafloor sediment. *Proc. Natl. Acad. Sci. U.S.A.* **114**, 2940–2945 (2017).

16. W. D. Orsi, V. P. Edgcomb, G. D. Christman, J. F. Biddle, Gene expression in the deep biosphere. *Nature* **499**, 205–208 (2013).

17. E. R. Estes, R. Pockalny, S. D'Hondt, F. Inagaki, Y. Morono, R. W. Murray, D. Nordlund, A. J. Spivack, S. D. Wankel, N. Xiao, C. M. Hansel, Persistent organic matter in oxic subseafloor sediment. *Nat. Geosci.* **12**, 126–131 (2019).

18. D. Canfield, E. Kristensen, B. Thamdrup, *Advances in Marine Biology: Aquatic Geomicrobiology* (Academic Press, 2005).

19. B. B. Jørgensen, Mineralization of organic matter in the sea bed—The role of sulphate reduction. *Nature* **296**, 643–645 (1982).

20. T. K. Tromp, P. Van Cappellen, R. M. Key, A global model for the early diagenesis of organic carbon and organic phosphorus in marine sediments. *Geochim. Cosmochim. Acta* **59**, 1259–1284 (1995).

21. B. B. Jørgensen, S. Kasten, Sulfur cycling and methane oxidation, in *Marine Geochemistry*, H. D. Schulz, M. Zabel, Eds. (Springer Berlin Heidelberg, 2006), pp. 271–309.

22. K. H. Nealson, P. G. Conrad, Life: Past, present and future. *Philos. Trans. R. Soc. Lond. B Biol. Sci.* **354**, 1923–1939 (1999).

23. C. B. Field, M. J. Behrenfeld, J. T. Randerson, P. Falkowski, Primary production of the biosphere: Integrating terrestrial and oceanic components. *Science* **281**, 237–240 (1998).

24. E. Marschall, M. Jogler, U. Henßge, J. Overmann, Large-scale distribution and activity patterns of an extremely low-light-adapted population of green sulfur bacteria in the Black Sea. *Environ. Microbiol.* **12**, 1348–1362 (2010).

25. L. Tijhuis, M. C. Van Loosdrecht, J. J. Heijnen, A thermodynamically based correlation for maintenance Gibbs energy requirements in aerobic and anaerobic chemotrophic growth. *Biotechnol. Bioeng.* **42**, 509–519 (1993).

26. F. Beulig, H. Røy, C. Glombitza, B. B. Jørgensen, Control on rate and pathway of anaerobic organic carbon degradation in the seabed. *Proc. Natl. Acad. Sci. U.S.A.* **115**, 367–372 (2018).

27. T. M. McCollom, J. P. Amend, A thermodynamic assessment of energy requirements for biomass synthesis by chemolithoautotrophic micro-organisms in oxic and anoxic environments. *Geobiology* **3**, 135–144 (2005).

28. M. A. Lever, K. L. Rogers, K. G. Lloyd, J. Overmann, B. Schink, R. K. Thauer, T. M. Hoehler, B. B. Jørgensen, Life under extreme energy limitation: A synthesis of laboratory- and field-based investigations. *FEMS Microbiol. Rev.* **39**, 688–728 (2015).

29. S. Arndt, B. B. Jørgensen, D. E. LaRowe, J. J. Middelburg, R. D. Pancost, P. Regnier, Quantifying the degradation of organic matter in marine sediments: A review and synthesis. *Earth Sci. Rev.* **123**, 53–86 (2013).

30. J. A. Bradley, J. P. Amend, D. E. LaRowe, Necromass as a limited source of energy for microorganisms in marine sediments. *J. Geophys. Res. Biogeosci.* **123**, 577–590 (2018).

31. S. Braun, S. S. Mhatre, M. Jaussi, H. Røy, K. U. Kjeldsen, C. Pearce, M.-S. Seidenkrantz, B. B. Jørgensen, B. A. Lomstein, Microbial turnover times in the deep seabed studied by amino acid racemization modelling. *Sci. Rep.* **7**, 5680 (2017).

32. Y. M. Bar-On, R. Phillips, R. Milo, The biomass distribution on Earth. *Proc. Natl. Acad. Sci. U.S.A.* **115**, 6506–6511 (2018).

33. C. Magnabosco, L.-H. Lin, H. Dong, M. Bomberg, W. Ghiorse, H. Stan-Lotter, K. Pedersen, T. L. Kieft, E. van Heerden, T. C. Onstott, The biomass and biodiversity of the continental subsurface. *Nat. Geosci.* **11**, 707–717 (2018).

34. S. E. Jones, J. T. Lennon, Dormancy contributes to the maintenance of microbial diversity. *Proc. Natl. Acad. Sci. U.S.A.* **107**, 5881–5886 (2010).

35. J. T. Lennon, S. E. Jones, Microbial seed banks: The ecological and evolutionary implications of dormancy. *Nat. Rev. Microbiol.* **9**, 119–130 (2011).

36. D. E. LaRowe, S. Arndt, J. A. Bradley, E. Burwicz, A. W. Dale, J. P. Amend, Organic carbon and microbial activity in marine sediments on a global scale throughout the Quaternary. *EarthArXiv* 10.31223/osf.io/fj5tz (2020).

37. K. Wallmann, E. Pinero, E. Burwicz, M. Haeckel, C. Hensen, A. Dale, L. Ruepke, The global inventory of methane hydrate in marine sediments: A theoretical approach. *Energies* **5**, 2449–2498 (2012).

38. R. A. Berner, Early diagenesis: A theoretical approach, in *Princeton Series in Geochemistry* (Princeton Univ. Press, 1980).

39. B. P. Boudreau, *Diagenetic Models and Their Implementation: Modelling Transport and Reactions in Aquatic Sediments* (Springer, 1997), vol. 171.

40. L. F. Athy, Density, porosity, and compaction of sedimentary rocks. *Am. Assoc. Pet. Geol. Bull.* **14**, 1–24 (1930).

41. B. P. Boudreau, B. R. Ruddick, On a reactive continuum representation of organic matter diagenesis. *Am. J. Sci.* **291**, 507–538 (1991).

42. R. Aris, Prolegomena to the rational analysis of systems of chemical reactions II. Some addenda. *Arch. Ration. Mech. Anal.* **27**, 356–364 (1968).

43. T. C. Ho, R. Aris, On apparent second-order kinetics. *AIChE J.* **33**, 1050–1051 (1987).

44. A. W. Dale, L. Nickelsen, F. Scholz, C. Hensen, A. Oschlies, K. Wallmann, A revised global estimate of dissolved iron fluxes from marine sediments. *Global Biogeochem. Cycles* **29**, 691–707 (2015).

45. K. Seiter, C. Hensen, J. Schröter, M. Zabel, Organic carbon content in surface sediments—Defining regional provinces. *Deep Sea Res. Part I Oceanogr. Res. Pap.* **51**, 2001–2026 (2004).

46. E. A. Romankevich, A. A. Vetrov, V. I. Peresypkin, Organic matter of the World Ocean. *Russ. Geol. Geophys.* **50**, 299–307 (2009).

47. E. B. Burwicz, L. H. Rüpke, K. Wallmann, Estimation of the global amount of submarine gas hydrates formed via microbial methane formation based on numerical reaction-transport modeling and a novel parameterization of Holocene sedimentation. *Geochim. Cosmochim. Acta* **75**, 4562–4576 (2011).

48. J. J. Middelburg, K. Soetaert, P. M. J. Herman, Empirical relationships for use in global diagenetic models. *Deep Sea Res. Part I Oceanogr. Res. Pap.* **44**, 327–344 (1997).

49. T. Hantschel, A. I. Kauerauf, *Fundamentals of Basin and Petroleum Systems Modeling* (Springer-Verlag, 2009).

50. R. N. Glud, Oxygen dynamics of marine sediments. *Mar. Biol. Res.* **4**, 243–289 (2008).

51. R. N. Glud, J. K. Gundersen, O. Holby, Benthic in situ respiration in the upwelling area off central Chile. *Mar. Ecol. Prog. Ser.* **186**, 9–18 (1999).

52. M. Egger, N. Riedinger, J. M. Mogollón, B. B. Jørgensen, Global diffusive fluxes of methane in marine sediments. *Nat. Geosci.* **11**, 421–425 (2018).

53. D. Hülse, S. Arndt, S. Daines, P. Regnier, A. Ridgwell, OMEN-SED 1.0: A novel, numerically efficient organic matter sediment diagenesis module for coupling to Earth system models. *Geosci. Model Dev.* **11**, 2649–2689 (2018).

54. M. Thullner, A. W. Dale, P. Regnier, Global-scale quantification of mineralization pathways in marine sediments: A reaction-transport modeling approach. *Geochem. Geophys. Geosyst.* **10**, Q10012 (2009).

55. R. J. Parkes, G. Webster, B. A. Cragg, A. J. Weightman, C. J. Newberry, T. G. Ferdelman, J. Kallmeyer, B. B. Jørgensen, I. W. Aiello, J. C. Fry, Deep sub-seafloor prokaryotes stimulated at interfaces over geological time. *Nature* **436**, 390–394 (2005).

56. J. J. Middelburg, L. A. Levin, Coastal hypoxia and sediment biogeochemistry. *Biogeosciences* **6**, 1273–1293 (2009).

57. P. Van Cappellen, Y. Wang, Cycling of iron and manganese in surface sediments: A general theory for the coupled transport and reaction of carbon, oxygen, nitrogen, sulfur, iron, and manganese. *Am. J. Sci.* **296**, 197–243 (1996).

58. D. E. LaRowe, P. Van Cappellen, Degradation of natural organic matter: A thermodynamic analysis. *Geochim. Cosmochim. Acta* **75**, 2030–2042 (2011).

59. D. E. LaRowe, J. P. Amend, Catabolic rates, population sizes and doubling/replacement times of microorganisms in natural settings. *Am. J. Sci.* **315**, 167–203 (2015).

60. H. C. Helgeson, D. H. Kirkham, G. C. Flowers, Theoretical prediction of the thermodynamic behavior of aqueous electrolytes by high pressures and temperatures: IV. Calculation of activity coefficients, osmotic coefficients, and apparent molal and standard and relative partial molal properties to 600°C and 5 kb. *Am. J. Sci.* **281**, 1249–1516 (1981).

61. J. C. Tanger IV, H. C. Helgeson, Calculation of the thermodynamic and transport properties of aqueous species at high pressures and temperatures: Revised equations of state for the standard partial molal properties of ions and electrolytes. *Am. J. Sci.* **288**, 19–98 (1988).

62. E. L. Shock, E. H. Oelkers, J. W. Johnson, D. A. Sverjensky, H. C. Helgeson, Calculation of the thermodynamic properties of aqueous species at high pressures and temperatures. Effective electrostatic radii, dissociation constants and standard partial molal properties to 1000 °C and 5 kbar. *J. Chem. Soc. Faraday Trans.* **88,** 803–826 (1992).

63. J. W. Johnson, E. H. Oelkers, H. C. Helgeson, SUPCRT92: A software package for calculating the standard molal thermodynamic properties of minerals, gases, aqueous species, and reactions from 1 to 5000 bar and 0 to 1000°C. *Comput. Geosci.* **18**, 899–947 (1992).

64. E. L. Shock, H. C. Helgeson, Calculation of the thermodynamic and transport properties of aqueous species at high pressures and temperatures: Correlation algorithms for ionic species and equation of state predictions to 5 kb and 1000°C. *Geochim. Cosmochim. Acta* **52**, 2009–2036 (1988).

65. E. L. Shock, H. C. Helgeson, D. A. Sverjensky, Calculation of the thermodynamic and transport properties of aqueous species at high pressures and temperatures: Standard partial molal properties of inorganic neutral species. *Geochim. Cosmochim. Acta* **53**, 2157–2183 (1989).

66. E. L. Shock, H. C. Helgeson, Calculation of the thermodynamic and transport properties of aqueous species at high pressures and temperatures: Standard partial molal properties of organic species. *Geochim. Cosmochim. Acta* **54**, 915–945 (1990).

67. D. A. Sverjensky, E. L. Shock, H. C. Helgeson, Prediction of the thermodynamic properties of aqueous metal complexes to 1000°C and 5 kb. *Geochim. Cosmochim. Acta* **61**, 1359–1412 (1997).

68. M. D. Schulte, E. L. Shock, R. H. Wood, The temperature dependence of the standard-state thermodynamic properties of aqueous nonelectrolytes. *Geochim. Cosmochim. Acta* **65**, 3919–3930 (2001).

69. H. C. Helgeson, Thermodynamics of hydrothermal systems at elevated temperatures and pressures. *Am. J. Sci.* **267**, 729–804 (1969).

70. B. B. Jørgensen, R. J. Parkes, Role of sulfate reduction and methane production by organic carbon degradation in eutrophic fjord sediments (Limfjorden, Denmark). *Limnol. Oceanogr.* **55**, 1338–1352 (2010).

71. A. Vion, L. Menot, Continental margins between 140m and 3500m depth (2009).

72. A. D. Steen, A. Crits-Christoph, P. Carini, K. M. DeAngelis, N. Fierer, K. G. Lloyd, J. C. Thrash, High proportions of bacteria and archaea across most biomes remain uncultured. *ISME J.* **13**, 3126–3130 (2019).

73. L. A. Zinke, C. Glombitza, J. T. Bird, H. Røy, B. B. Jørgensen, K. G. Lloyd, J. P. Amend, B. K. Reese, Microbial organic matter degradation potential in Baltic Sea sediments influenced by depositional conditions and in situ geochemistry. *Appl. Environ. Microbiol.* **85**, e02164-18 (2019).

74. W. D. Orsi, T. A. Richards, W. R. Francis, Predicted microbial secretomes and their target substrates in marine sediment. *Nat. Microbiol.* **3**, 32–37 (2018).

75. B. J. Baker, C. S. Lazar, A. P. Teske, G. J. Dick, Genomic resolution of linkages in carbon, nitrogen, and sulfur cycling among widespread estuary sediment bacteria. *Microbiome* **3**, 14 (2015).

76. I. P. G. Marshall, S. M. Karst, P. H. Nielsen, B. B. Jørgensen, Metagenomes from deep Baltic Sea sediments reveal how past and present environmental conditions determine microbial community composition. *Mar. Genomics* **37**, 58–68 (2018).

77. L. A. Zinke, M. M. Mullis, J. T. Bird, I. P. G. Marshall, B. B. Jørgensen, K. G. Lloyd, J. P. Amend, B. K. Reese, Thriving or surviving? Evaluating active microbial guilds in Baltic Sea sediment. *Environ. Microbiol. Rep.* **9**, 528–536 (2017).

78. W. D. Orsi, Ecology and evolution of seafloor and subseafloor microbial communities. *Nat. Rev. Microbiol.* **16**, 671–683 (2018).

79. J. F. Biddle, J. S. Lipp, M. A. Lever, K. G. Lloyd, K. B. Sørensen, R. Anderson, H. F. Fredricks, M. Elvert, T. J. Kelly, D. P. Schrag, M. L. Sogin, J. E. Brenchley, A. Teske, C. H. House, K.-U. Hinrichs, Heterotrophic Archaea dominate sedimentary subsurface ecosystems off Peru. *Proc. Natl. Acad. Sci. U.S.A.* **103**, 3846–3851 (2006).

80. S. E. Ruff, J. F. Biddle, A. P. Teske, K. Knittel, A. Boetius, A. Ramette, Global dispersion and local diversification of the methane seep microbiome. *Proc. Natl. Acad. Sci. U.S.A.* **112**, 4015–4020 (2015).

81. B. K. Reese, L. A. Zinke, M. S. Sobol, D. E. LaRowe, B. N. Orcutt, X. Zhang, U. Jaekel, F. Wang, T. Dittmar, D. Defforey, B. Tully, A. Paytan, J. B. Sylvan, J. P. Amend, K. J. Edwards, P. Girguis, Nitrogen cycling of active bacteria within oligotrophic sediment of the mid-atlantic ridge flank. *Geomicrobiol. J.* **35**, 468–483 (2018).

82. B. J. Tully, J. F. Heidelberg, Potential mechanisms for microbial energy acquisition in oxic deep- sea sediments. *Appl. Environ. Microbiol.* **82**, 4232–4243 (2016).

83. A. Durbin, A. Teske, Microbial diversity and stratification of South Pacific abyssal marine sediments. *Environ. Microbiol.* **13**, 3219–3234 (2011).

84. J. Chun, A. Oren, A. Ventosa, H. Christensen, D. R. Arahal, M. S. da Costa, A. P. Rooney, H. Yi, X.-W. Xu, S. De Meyer, M. E. Trujillo, Proposed minimal standards for the use of genome data for the taxonomy of prokaryotes. *Int. J. Syst. Evol. Microbiol.* **68**, 461–466 (2018).

85. A. Tkacz, M. Hortala, P. S. Poole, Absolute quantitation of microbiota abundance in environmental samples. *Microbiome* **6**, 110 (2018).

86. C. L. Myers, D. R. Barrett, M. A. Hibbs, C. Huttenhower, O. G. Troyanskaya, Finding function: Evaluation methods for functional genomic data. *BMC Genomics* **7**, 187 (2006).

87. J. M. Janda, S. L. Abbott, 16S rRNA gene sequencing for bacterial identification in the diagnostic laboratory: Pluses, perils, and pitfalls. *J. Clin. Microbiol.* **45**, 2761–2764 (2007).

88. K. H. Nealson, Sediment bacteria: Who's there, what are they doing, and what's new? *Annu. Rev. Earth Planet. Sci.* **25**, 403–434 (1997).

89. C. Arnosti, C. Bell, D. L. Moorhead, R. L. Sinsabaugh, A. D. Steen, M. Stromberger, M. Wallenstein, M. N. Weintraub, Extracellular enzymes in terrestrial, freshwater, and marine environments: Perspectives on system variability and common research needs. *Biogeochemistry* **117**, 5–21 (2014).

90. K. G. Lloyd, A. D. Steen, J. Ladau, J. Yin, L. Crosby, Phylogenetically novel uncultured microbial cells dominate Earth microbiomes. *mSystems* **3**, e00055-18 (2018).

91. D. J. Burdige, K. G. Gardner, Molecular weight distribution of dissolved organic carbon in marine sediment pore waters. *Mar. Chem.* **62**, 45–64 (1998).

92. C. A. Fox, H. A. Abdulla, D. J. Burdige, J. P. Lewicki, T. Komada, Composition of dissolved organic matter in pore waters of anoxic marine sediments analyzed by ^{1}H nuclear magnetic resonance spectroscopy. *Front. Mar. Sci.* **5**, 172 (2018).

93. D. E. LaRowe, E. Burwicz, S. Arndt, A. W. Dale, J. P. Amend, Temperature and volume of global marine sediments. *Geology* **45**, 275–278 (2017).

94. J. M. Mogollón, A. W. Dale, H. Fossing, P. Regnier, Timescales for the development of methanogenesis and free gas layers in recently-deposited sediments of Arkona Basin (Baltic Sea). *Biogeosciences* **9**, 1915–1933 (2012).

95. K. Wallmann, G. Aloisi, M. Haeckel, A. Obzhirov, G. Pavlova, P. Tishchenko, Kinetics of organic matter degradation, microbial methane generation, and gas hydrate formation in anoxic marine sediments. *Geochim. Cosmochim. Acta* **70**, 3905–3927 (2006).

96. J. J. Middelburg, A simple rate model for organic matter decomposition in marine sediments. *Geochim. Cosmochim. Acta* **53**, 1577–1581 (1989).

97. J. T. Westrich, R. A. Berner, The role of sedimentary organic-matter in bacterial sulfate reduction: The G Model Tested. *Limnol. Oceanogr.* **29**, 236–249 (1984).

98. M. Marquardt, C. Hensen, E. Piñero, K. Wallmann, M. Haeckel, A transfer function for the prediction of gas hydrate inventories in marine sediments. *Biogeosciences* **7**, 2925–2941 (2010).

99. R. N. Glud, J. K. Gundersen, B. B. Jørgensen, N. P. Revsbech, H. D. Schulz, Diffusive and total oxygen uptake of deep-sea sediments in the eastern South Atlantic Ocean: In situ and laboratory measurements. *Deep Sea Res. Part I Oceanogr. Res. Pap.* **41**, 1767–1788 (1994).

100. R. N. Glud, J. K. Gundersen, H. Røy, B. B. Jørgensen, Seasonal dynamics of benthic O_2 uptake in a semienclosed bay: Importance of diffusion and faunal activity. *Limnol. Oceanogr.* **48**, 1265–1276 (2003).

101. R. N. Glud, O. Holby, F. Hoffmann, D. Canfield, Benthic mineralization and exchange in Arctic sediments (Svalbard, Norway). *Mar. Ecol. Prog. Ser.* **173**, 237–251 (1998).

102. F. Wenzhöfer, O. Holby, O. Kohls, Deep penetrating benthic oxygen profiles measured in situ by oxygen optodes. *Deep Sea Res. Part I Oceanogr. Res. Pap.* **48**, 1741–1755 (2001).

103. F. Wenzhöfer, M. Adler, O. Kohls, C. Hensen, B. Strotmann, S. Boehme, H. D. Schulz, Calcite dissolution driven by benthic mineralization in the deep-sea: In situ measurements of Ca^{2+}, pH, pCO_2 and O_2. *Geochim. Cosmochim. Acta* **65**, 2677–2690 (2001).

104. R. A. Jahnke, S. R. Emerson, C. E. Reimers, J. Schuffert, K. Ruttenberg, D. Archer, Benthic recycling of biogenic debris in the eastern tropical Atlantic Ocean. *Geochim. Cosmochim. Acta* **53**, 2947–2960 (1989).

105. F. Wenzhöfer, R. N. Glud, Benthic carbon mineralization in the Atlantic: A synthesis based on in situ data from the last decade. *Deep Sea Res. Part I Oceanogr. Res. Pap.* **49**, 1255–1279 (2002).

106. U. Witte, N. Aberle, M. Sand, F. Wenzhöfer, Rapid response of a deep-sea benthic community to POM enrichment: An in situ experimental study. *Mar. Ecol. Prog. Ser.* **251**, 27–36 (2003).

107. U. Witte, F. Wenzhöfer, S. Sommer, A. Boetius, P. Heinz, N. Aberle, M. Sand, A. Cremer, W.-R. Abraham, B. B. Jørgensen, O. Pfannkuche, In situ experimental evidence of the fate of a phytodetritus pulse at the abyssal sea floor. *Nature* **424**, 763–766 (2003).

108. K. S. Black, G. R. Fones, O. C. Peppe, H. A. Kennedy, I. Bentaleb, An autonomous benthic lander:: Preliminary observations from the UK BENBO thematic programme. *Cont. Shelf Res.* **21**, 859–877 (2001).

109. R. N. Glud, H. Stahl, P. Berg, F. Wenzhöfer, K. Oguri, H. Kitazato, In situ microscale variation in distribution and consumption of 2: A case study from a deep ocean margin sediment (Sagami Bay, Japan). *Limnol. Oceanogr.* **54**, 1–12 (2009).

110. P. Giordani, W. Helder, E. Koning, S. Miserocchi, R. Danovaro, A. Malaguti, Gradients of benthic–pelagic coupling and carbon budgets in the Adriatic and Northern Ionian Sea. *J. Mar. Syst.* **33–34**, 365–387 (2002).

111. B. Lansard, C. Rabouille, L. Denis, C. Grenz, In situ oxygen uptake rates by coastal sediments under the influence of the Rhône River (NW Mediterranean Sea). *Cont. Shelf Res.* **28**, 1501–1510 (2008).

112. B. Lansard, C. Rabouille, L. Denis, C. Grenz, Benthic remineralization at the land–ocean interface: A case study of the Rhône River (NW Mediterranean Sea). *Estuar. Coast. Shelf Sci.* **81**, 544–554 (2009).

113. O. Sachs, E. J. Sauter, M. Schlüter, M. M. R. van der Loeff, K. Jerosch, O. Holby, Benthic organic carbon flux and oxygen penetration reflect different plankton provinces in the Southern Ocean. *Deep Sea Res. Part I Oceanogr. Res. Pap.* **56**, 1319–1335 (2009).

114. D. E. Canfield, B. B. Jørgensen, H. Fossing, R. Glud, J. Gundersen, N. B. Ramsing, B. Thamdrup, J. W. Hansen, L. P. Nielsen, P. O. J. Hall, Pathways of organic carbon oxidation in three continental margin sediments. *Mar. Geol.* **113**, 27–40 (1993).
